# A peleg modeling of water absorption in cold plasma-treated Chickpea (*Cicer arietinum L.*) cultivars

**DOI:** 10.1038/s41598-023-33802-y

**Published:** 2023-05-15

**Authors:** F. L. Pathan, A. M. Trimukhe, R. R. Deshmukh, U. S. Annapure

**Affiliations:** 1 Department of Food Engineering and Technology, Institute of Chemical Technology, N.P. Marg, Matunga, Mumbai (E) 400019 India; 2 Department of Physics, Institute of Chemical Technology, N.P. Marg, Matunga, Mumbai (E) 400019 India; 3 Institute of Chemical Technology, Marathwada Campus, Aurangabad Road, Jalana, 431213 India

**Keywords:** Biochemistry, Biological techniques, Biophysics, Physics

## Abstract

Plasma processing appears to be the mainstay of food preservation in the present day due to its effectiveness in controlling microorganisms at low temperatures. Legumes are usually soaked before cooking. Six chickpea varieties (*Kripa, Virat, Vishal, Vijay, Digvijay, and Rajas*) were soaked in distilled water at room temperature, and Peleg model was fitted after plasma treatment. Cold plasma treatment was used at 40, 50 and 60 Watt with exposure times of 10, 15 and 20 min. K_1_ (Peleg rate constant) consistently decreased from 32.3 to 4.3 × 10^–3^ (h % − 1) for all six chickpea cultivars, indicating an increased water absorption rate with increasing plasma power and treatment time. It was lowest in 60 W 20 min plasma treatment in *Virat* cultivar. K_2_ (Peleg capacity constant) ranged from 9.4 to 12 × 10^–3^ (h % − 1) for all six chickpea cultivars. Thus, plasma treatment showed no effect on water uptake capacity (K_2_), as it did not increase or decrease consistently with increasing plasma power and treatment time. Fitting the Peleg model successfully revealed the correlation between the water absorption of chickpea cultivars. The model fit ranged from *R*^2^ ≥ 0.9873 to 0.9981 for all six chickpea cultivars.

## Introduction

Chickpea (*Cicer arietinum L*.) is the third most important legume in the world in terms of consumption. There are two main types of chickpeas, *Desi* and *Kabuli*. The latter has a thin, white seed coat. The former has a thick, coloured seed coat. *Kabuli* is larger than *Desi*^1^. They are a source of energy, vitamins and minerals, as well as economical sources of protein. Chickpeas have been shown to reduce cardiovascular disease, diabetes, obesity and cancer^2^.

In the case of legumes, soaking must be carried out prior to their use and processing. For example, the extrusion of pulses should be done after soaking them in water for 16 h to improve their nutritional value ^3^. Gelatinisation is achieved by soaking starch^7^. Processes such as soaking, scorching, etc. are used to increase protein digestibility, mineral bioavailability and thus the nutritional value of foods^4,5^. The water absorption rates and water absorption capacities of grains under different soaking conditions have been investigated^6–9^. The water absorbed by pulses during soaking affects subsequent processing and thus the quality of the final product^10^. The water absorption of seeds is determined by the time of soaking and the temperature of the soaking water. During soaking, water slowly enters the seeds, eventually reaching a constant moisture content^11^. The processing operations like cooking, germination, milling, and the quality of the final product are dependent on the water sorption during the soaking process. Therefore, the kinetics of water absorption in pulses during soaking has been studied^10,12–16^. Considerable efforts have been made by scientists under this topic. However, plasma-treated chickpea has not yet been studied in detail. In view of the present scenario of plasma treatment in food processing, it will soon be the most common treatment; therefore, efforts are being made to study it.

Water absorption has been studied using various theoretical and empirical approaches. In some cases, empirical models tended to be preferred because they seemed relatively easy to use^17^. Peleg has used a two-parameter empirical model which has been successfully used to describe the water sorption behaviour of chickpeas, field peas, kidney beans, pigeon peas, peanuts and soya beans^12–16^. It is the most popular model because of its simplicity and has also been used to study sorption processes in wheat, rice, sago starch, papaya and dasheen leaves^18–23^.

Sir William Crookes was the first to describe plasma^24^. It is the fourth state of matter which is partially or fully ionised state of gas^25^. Cold plasma is growing rapidly and is starting to show potential for sterilising surfaces of food, packaging and fresh produce^26–28^. Endospore-forming bacteria and spores with durable coat layers, such as *Clostridia* and *Bacillus subtilis*, are comparatively resistant to conventional sterilisation methods. Cold plasma offers a practical solution and appears to be a fast, inexpensive and environmentally friendly tool to improve crop yields^29,30^. Hydrogenation of edible oils, removal of anti-nutritional factors, alleviation of food allergies, modification of seed germination and wastewater management are other areas of its use^31^. Cold plasma is used to control pollutants and plant growth and as an alternative to pesticides and fertilisers^32^.

Fresh spinach leaves treated with plasma showed discolouration after 24 h cold storage^33,34^. It can inactivate hypertonic yeast (*Zygosaccharomyces rouxii*) in apple juice^35^. It has also been studied to reduce cooking time^36^. Soaking plasma treated chickpeas (*Cicer arientinum*) was investigated by Pathan et al. (2021). This study reported soaking plasma-treated samples in distilled water and 1% sodium bicarbonate solution at room temperature^37^. Cold plasma has also been successfully used to protect chickpeas against the storage pest *Callosobruchus chinensis* (Chrysomelidae: Bruchinae) for approximately four years^38^.

After cold plasma treatment the seed surface oxygen-containing functional group’s intensity increases resulting in increased surface wettability through the surface oxidation process^39^. The scanning electron micrographs of the plasma-treated surface show erosion in an uppermost layer in the seed coat, viz. the upper epidermis^33^. Cold plasma pretreatment can reduce soaking and cooking times of legume seeds. The aim of the present research was to investigate the suitability of the Peleg model for predicting the water absorption behaviour of chickpea cultivars treated with cold plasma and to study the characterisation of the equation constants while determining the appropriate conditions for the soaking process.

## Materials and methods

### Materials

The chickpea samples were procured from the Pulses Improvement Project, MPKV, Rahuri, India. The names of the cultivars procured were *Kripa, Virat* (Kabuli type) *Vishal, Vijay, Digvijay,* and *Rajas* (Desi type). The Initial moisture content of these samples was determined by using the AOAC (2002) method^40^. Which was 8.4, 8.2, 10, 10.10, 9.3, and 9.1% on a dry basis for all the chickpea cultivars, respectively. These samples were adequately cleaned and kept in a zip-lock pouch before and after plasma treatment.

### Methods

#### Plasma treatment of chickpea cultivars

**Figure 1:**
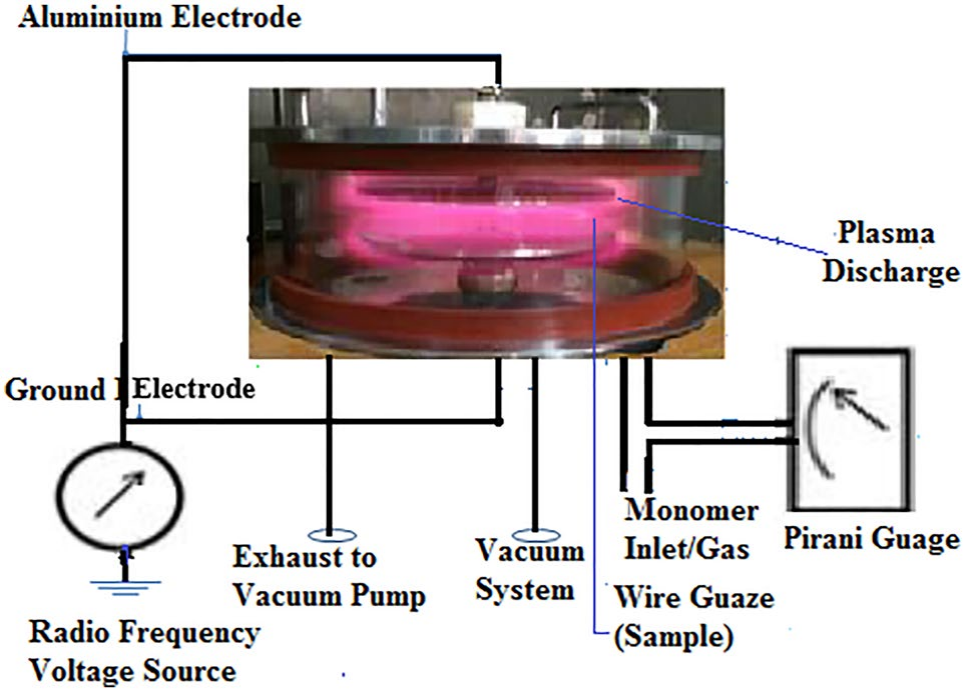
Schematic experimental setup for low pressure plasma system.

An in-house built low-pressure glow discharge plasma with bell-jar symmetry was employed for plasma treatment. The reactor walls were made up of Pyrex glass with 3 mm of thickness, a height of 120 mm, and an internal diameter of 300 mm; the material of the base and the opening lid was stainless steel. The electrodes were made of aluminium which has a 20 cm diameter. The electrodes were connected through the Wilson seals on these plates. The base plate had ports to connect the gas/monomer reservoir, Pirani gauge, vacuum pump, air admittance valve, etc. The electrode distance inside the reactor was maintained at 3 cm during all the plasma treatments. The system was capacitively coupled with a radiofrequency power source having a frequency of 13.56 MHz. The system pressure was initially achieved at 0.05 mBar with samples in the system by using HHV vacuum pump ED-20, and the working pressure was adjusted to the optimised value of 0.5 mBar. The plasma glow was observed (Fig. 1), and treatment on chickpea cultivars was performed at 40, 50, and 60 W, each having an exposure time of 10, 15, and 20 min.

#### Soaking test

Chickpea samples (8 g) were poured into a beaker containing 50 ml of distilled water at room temperature. During soaking, samples were removed from the beaker at the interval of 1 h. and placed on a filter paper until they lost surface moisture. All samples were weighed and returned to the beaker. The beaker was topped up with distilled water. A stopwatch in a cell phone and a precision electronic balance (Model CAH 323, accuracy ± 0.001 g India) were used, respectively, to record soaking time and weigh the sample before and after immersion of chickpea. Experiments were terminated at equilibrium moisture content after a sample mass showed a change of less than 0.001 g in consecutive 1 h of soaking. All the soaking experiments were performed in triplicate. The per cent moisture absorbed was calculated ^7,10,37^.

1 $${\text{Wa}} = \frac{{{\text{Wf }} - {\text{Wi}}}}{{{\text{Wi}}}} {\text{X }}100$$

Where Wa is water absorption (d.b. %), Wf is the weight of seeds after immersion (g) and Wi is the weight of seeds before immersion (g).

### Analysis of soaking data and soaking model

A low amount of water was absorbed by seeds before 3 h, the recorded data for this period were not used for fitting the Peleg model. Also, the control samples did not reach the equilibrium moisture content in 9 h. hence their data of soaking after this was not considered for Peleg Model fitting.

### Modelling water absorption of plasma-treated chickpea cultivars

Peleg proposed an equation which is as follows:

2 $${\mathbf{M}}_{{\mathbf{t}}} = {\mathbf{M}}_{0} \pm \frac{{\mathbf{t}}}{{{\mathbf{K}}_{1} + {\mathbf{K}}_{2} {\mathbf{t}}}}\user2{ }$$

Where M_t_ is moisture content at time t (% d. b.), M_o_ is initial moisture content (% d. b.), t is time (h), K_1_ and K_2_ are the Peleg rate (h%^−1^), and Peleg capacity constant (%^−1^) respectively. In Eq. (1), ‘ ± ’ becomes ‘+’ if the process is absorption and ‘−’ if the process is drying or desorption.

The rate of sorption (R) can be obtained from the first derivative of the Peleg equation:

3 $${\mathbf{R}} = \frac{{{\mathbf{dM}}}}{{{\mathbf{dt}}}} = \pm \frac{{{\mathbf{K}}_{1} }}{{\left( {{\mathbf{K}}_{1} + {\mathbf{K}}_{2} {\mathbf{t}}} \right)^{2} }}$$

The Peleg rate constant K_1_ relates to the sorption rate at the starting (Ro), i.e., R at t = to

4 $${\mathbf{R}}_{0} = \left. {\frac{{{\mathbf{dM}}}}{{{\mathbf{dt}}}}} \right|_{{{\mathbf{t}}_{0} }} = \pm \frac{1}{{{\mathbf{K}}_{1} }}$$

The Peleg capacity constant K_2_ relates to the maximum (or minimum) attainable moisture content. As Eq. (4) gives the relation between equilibrium moisture content (Me) and K_2_

5 $$\left. {\mathbf{M}} \right|_{{{\mathbf{t}}_{\infty } }} = {\mathbf{M}}_{{\mathbf{e}}} = {\mathbf{M}}_{0} \pm \frac{1}{{{\mathbf{K}}_{{\begin{array}{*{20}c} 2 \\ {} \\ \end{array} }} }}$$

e. Calculation of Coefficient of determination (R^2^) and Root Mean Square Error

To evaluate Peleg model forecasting, predicted data were plotted against test data for six studied varieties treated at 40, 50, and 60 W each for 10, 15, and 20 min and the coefficient of determination (R^2^) was determined by following Eq. (6):

6 $${\text{R}}^{2} = \frac{{\begin{array}{*{20}c} {} \\ {\mathop \sum \nolimits_{i = 1}^{n} [(M_{exp,i} - M_{\exp ave} )]^{2} - \mathop \sum \nolimits_{i = 1}^{n} [(M_{exp,i} - M_{pre,i} )]^{2} } \\ \end{array} }}{{\mathop \sum \nolimits_{i = 1}^{n} [(M_{exp,i} - M_{\exp ave} )]^{2} }}$$

Where Mexp, i is the i^th^ experimentally observed moisture content, Mpre, i is the i^th^ predicted moisture content, M exp ave is the average moisture content observed, and n is the number of observations.

The goodness of fit between the experimental and predicted water absorption values was calculated using the Root Mean Square Error (RMSE), as:

Root mean square error, %,

7 $${\text{Root}}\;{\text{mean}}\;{\text{square}}\;{\text{error}},\% ,\;RMSE = 100 { \times } \sqrt {\frac{1}{n} \mathop \sum \limits_{1}^{n} \left[ {\left( {M_{exp} - M_{pre} } \right)/M_{exp} } \right]^{2} }$$

Where Mexp, i is the ith experimentally observed moisture content, Mpre, is the ith predicted moisture content, M exp ave is the average moisture content observed and n is the number of observations.

### Statistical analysis

The findings were statistically analysed using SPSS (IBM statistical analysis version 19) and one-way ANOVA for the increase in moisture (%). All samples were analysed in triplicate. The significance between the samples was compared at *P* < 0.05, where the least significant difference was tested by the Post-hoc and Duncan tests. The averages from three different studies were presented in all of the findings. The testing of the Peleg model and the curve fitting was carried out in Microsoft Excel using the least-squares method.

### Research involving plants

The present study complies with relevant institutional, national, and international guidelines and legislation as per the IUCN Policy Statement on research involving species at risk of extinction and the Convention on international trade in endangered wild fauna and flora species.

### Ethical approval

This article does not contain any studies with human participants performed by any authors.

### Human and animal rights

The research did not involve human participants and/or animals.

## Results and discussion

### Water absorption characteristics of the legume seeds during soaking

**Figure 2:**
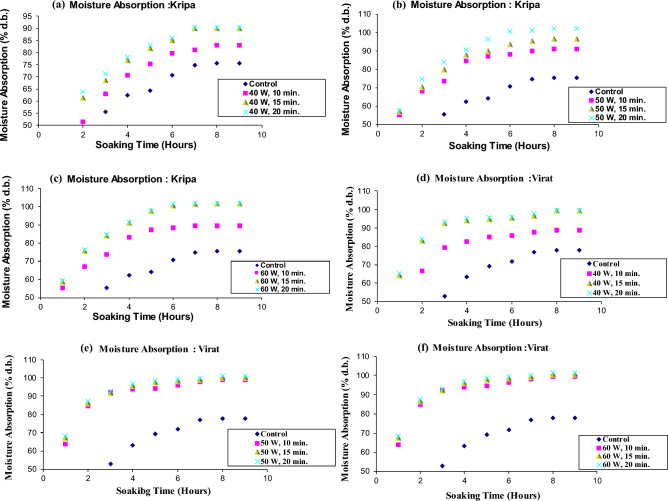

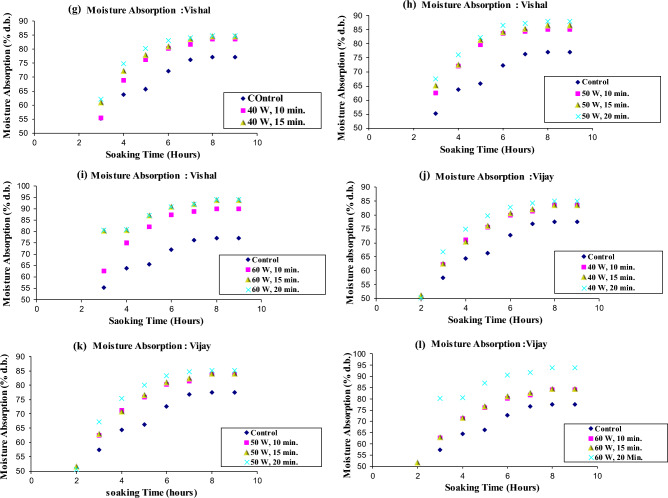

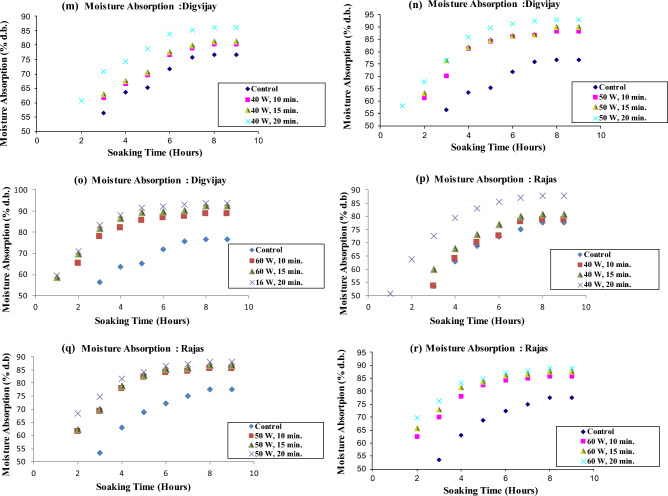
Moisture absorption curve of cold plasma treated six chickpea cultivars during soaking in distilled water.

The mean moisture content of cold plasma treated and untreated chickpea cultivars soaked in distilled water at ambient temperature are illustrated in Fig. 2, Table 1, and Supplementary Tables S1 and S2.

**Table 1:** Comparison of increase in moisture content of plasma treated and plasma untreated samples of six chickpea cultivars after soaking in distilled water. All the data are expressed as mean ± standard error. Means with the different superscript letters in a column differ significantly (*P* < 0.05).

Plasma treatment		Increase in moisture (% )					
Power (W)	Exposure time (min)	Kripa	Virat	Vishal	Vijay	Digvijay	Rajas
Control		75.65 ± 0.17^a^	77.75 ± 0.50^a^	77.063 ± 0.31^a^	77.50 ± 0.14^a^	76.59 ± 0.66^a^	77.51 ± 0.47^a^
40	10	82.90 ± 0.43^b^	88.68 ± 0.21^b^	83.61 ± 0.47^b^	83.56 ± 0.19^b^	80.43 ± 0.16^b^	78.57 ± 0.81^b^
	15	89.92 ± 0.28^c^	99.12 ± 0.26^c^	84.44 ± 0.61^c^	83.59 ± 0.26^b^	81.27 ± 0.13^c^	80.71 ± 0.73^c^
	20	90.53 ± 0.38^d^	99.78 ± 0.17^d^	84.77 ± 0.89^c^	84.83 ± 0.14^d^	86.14 ± 0.91^d^	87.69 ± 0.62f.
50	10	89.59 ± 0.98^c^	99.05 ± 0.35^c^	84.96 ± 0.10^d^	83.78 ± 0.26^c^	88.14 ± 0.99^e^	85.49 ± 0.70^d^
	15	96.53 ± 0.35^e^	100.16 ± 0.12^e^	86.52 ± 0.69^e^	84.00 ± 0.25f.	89.81 ± 0.83^ g^	86.68 ± 0.10^e^
	20	102.15 ± 0.10^ g^	101.17 ± 0.13^ g^	87.85 ± 0.60f.	85.21 ± 0.14^ g^	92.81 ± 0.42^ h^	87.87 ± 0.65^ g^
60	10	90.96 ± 0.54^d^	99.30 ± 0.44^c^	89.92 ± 0.79^ g^	83.93 ± 0.22^e^	88.64 ± 0.10f.	85.78 ± 0.73^d^
	15	101.57 ± 0.25f.	100.46 ± 0.72f.	93.85 ± 0.49^ h^	84.25 ± 0.11f.	92.26 ± 0.94^ h^	87.67 ± 0.81^ g^
	20	102.07 ± 0.28^ g^	101.42 ± 0.13^ g^	93.94 ± 0.45^i^	93.70 ± 0.83^ h^	93.64 ± 0.11^i^	88.80 ± 0.55^ h^

The moisture absorption curves reveal that moisture absorbed was much less in all the control samples than in all the plasma-treated chickpea cultivars, increasing from 40 watts 10 min to 60 watts 20 min. Thus, absorption curves showed that the water absorption rate increased with increasing plasma power and treatment time.

The percentage of water absorption was in the range of 75.65–77.75% and 78.57–102.07% for the control and plasma treated samples, respectively, during soaking in distilled water. A significant difference (*P* < 0.05) in moisture absorbed during distilled water soaking was found in samples exposed to 40, 50 and 60 W power for 10, 15 and 20 min in all chickpea cultivars. These results are in agreement with previous findings by researchers^37^. In the control samples, after soaking for 9 h, the moisture absorption was lowest in *Kripa* and highest in *Virat*. Previous researchers reported that water absorption values were significantly different (*P* < 0.05) in different chickpea cultivars. This was due to differences in morphological and physiological characteristics of chickpea cultivars grown in different regions. Our results do not agree with those reported results^41^. In our case, the chickpea's morphological and physiological properties did not differ as these were grown in the same place.

Among all the plasma treated samples of all the cultivars, the moisture absorption was the highest at the power of 60 W for a 20 min treatment during the soaking in distilled water, while it was the lowest at the power of 40 W for a 10 min treatment. The increase in moisture absorption was highest in *Kripa* at 60 W for 20 min treatment and lowest in *Rajas* at 40 W for 10 min treatment. The increase in water absorption with increase in plasma power and treatment time showed that plasma treatment may have eroded the surface of chickpea seeds and increased their surface energy. Cold plasma treatment and its effects on the germination of mung beans (*Vigna radiata*) have been studied^42^. Authors reported that the surface etching of mung beans is caused by the plasma species, which are responsible for increasing the conductivity of the seed coat and reducing the contact angle, making the surface more hydrophilic. The contact angle quantifies the wettability of a solid surface by a liquid. In the past, researchers observed scanning electron micrographs in black grams to confirm that plasma treatment caused surface etching and made the seed surface hydrophilic. This subsequently allowed easy absorption of water in black grams. They showed that the plasma treatment caused a decrease in the contact angle and an increase in the surface free energy^43^. The increased water absorption with increasing plasma power and treatment time in our case also confirmed that the rapid water absorption could be due to the surface etching of the plasma treated chickpea samples and also due to the reduced contact angle and increased surface free energy. Bormashenko et al., 2012, also reported that the wetting properties of seed surfaces could be modified by cold radiofrequency air plasma treatment for lentils (*Lens culinaris*), beans (*Phaseolus vulgaris*) and wheat (*Triticum, species* C9)^44^. The saturation moisture content of the bean varieties was reached at the same time^41,46^. However, in the case of the chickpea varieties, the saturation moisture content was comparatively shorter at higher water temperatures^41,45,46^. The increase in water diffusivity in the seeds may be responsible for this phenomenon. High temperatures could cause the seeds to soften and expand^41,45,46^. When the soaking temperature was closer to the seed gelatinising temperature, it had a higher moisture uptake rate^41,45,46^.

The water uptake rate in the present study was higher in the early soaking period and gradually decreased in all six chickpea cultivars later. At the 8^th^ and 9^th^ hours of soaking in distilled water, the cold plasma treated chickpea cultivars reached equilibrium. Cultivar *Virat* showed comparatively higher water uptake than *Digvijay, Rajas, Vijay, Kripa and Vishal* in decreasing order at each plasma power and exposure time.

### Modelling of the chickpea water absorption as a function of time

**Figure 3:**
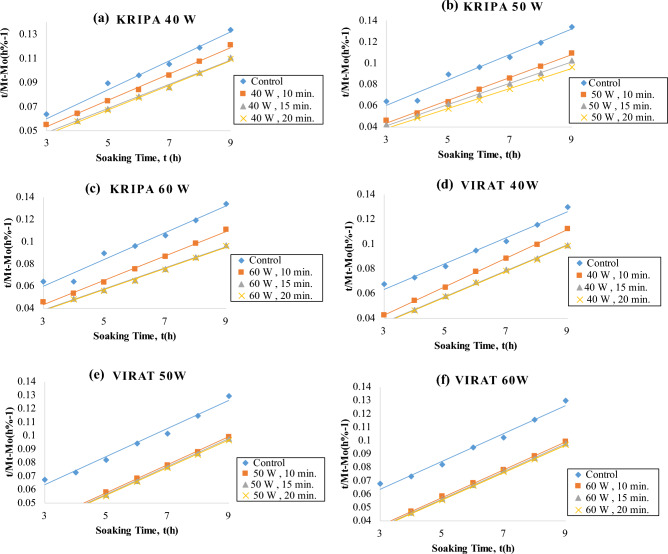

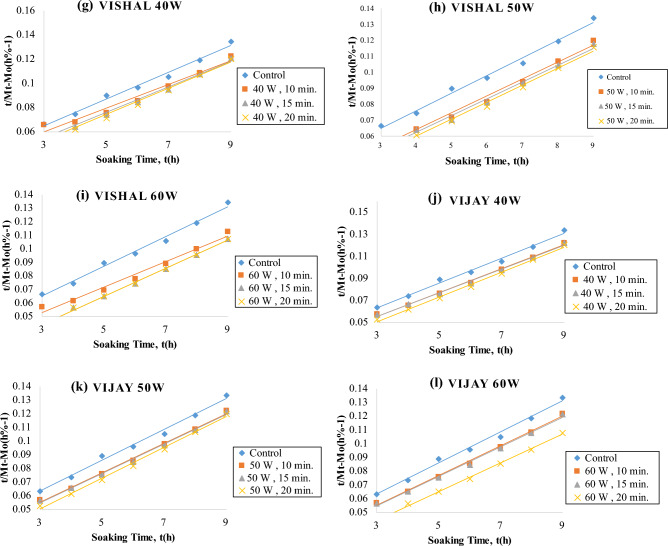

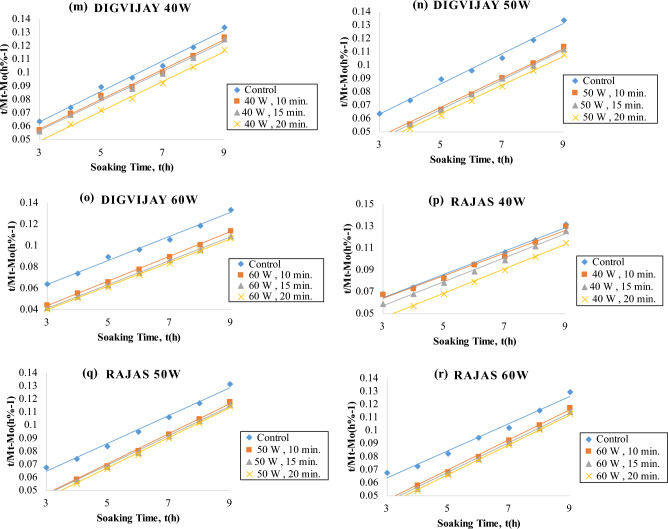
Fitting of the Peleg model to water absorption data of cold plasma treated six chickpea cultivars during soaking in distilled water.

The data up to t_0_ and t∞ were used for the determination of the goodness of fit of the Peleg model. The model fit resulted in R_2_ ≥ : 0.9981 for *Virat*, 0.9965 for *Digvijay*, 0.9953 for *Rajas*, 0.9950 for *Vijay*, 0.9941 for *Kripa* and 0.9873 for *Vishal* for the control samples. A typical fit is shown in Fig. 3 and the R_2_ and RMSE % values obtained from the fit of Eq. (2) are given in Table 2.

**Table 2:** Average Peleg constants and goodness of fit of Peleg model for water absorption of the untreated and cold plasma treated six chickpea cultivars in distilled water.

Plasma treatment		K_1_ × 10^–3^ (h % ^−1^)	K_2_ × 10^–3^ (%^−1^)	R^2^	RMSE (%)^a^
Plasma power (W)	Exposure time (min)				
Kripa					
Control	–	24.00	12.00	0.9723	0.084
40	10	20.60	10.90	0.9966	0.034
	15	18.80	10.00	0.9958	0.036
	20	16.60	10.10	0.9971	0.027
50	10	11.20	10.70	0.9960	0.062
	15	10.90	10.00	0.9988	0.027
	20	10.80	9.30	0.9971	0.037
60	10	10.20	10.00	0.9953	0.063
	15	10.00	9.50	0.9968	0.045
	20	9.80	9.40	0.9960	0.047
Virat					
Control	*–*	32.20	10.40	0.9855	0.064
40	10	7.50	11.50	0.9997	0.012
	15	5.00	10.50	0.9988	0.028
	20	4.60	10.40	0.9991	0.026
50	10	5.10	10.40	0.9994	0.023
	15	4.50	10.30	0.9999	0.015
	20	4.40	10.20	0.9999	0.014
60	10	5.10	10.40	0.9994	0.022
	15	4.50	10.30	0.9999	0.011
	20	4.30	10.20	0.9999	0.013

Plasma treatment		K_1_ × 10^–3^ (h % ^−1^)	K_2_ × 10^–3^ (%^−1^)	R^2^	RMSE (%)^a^
Plasma power(W)	Exposure Time (min)				
*Vishal*					
Control	–	31.80	11.00	0.9895	0.047
40	10	30.60	9.70	0.9693	0.010
	15	23.40	10.50	0.9885	0.069
	20	20.10	10.80	0.9840	0.098
50	10	24.60	10.60	0.9850	0.078
	15	20.60	10.50	0.9901	0.063
	20	17.80	10.60	0.9920	0.059
60	10	21.80	9.40	0.9810	0.083
	15	12.60	10.40	0.9972	0.045
	20	12.50	10.40	0.9972	0.045
Vijay					
Control	–	29.30	11.30	0.9912	0.045
40	10	22.80	10.80	0.9957	0.039
	15	22.80	10.80	0.9944	0.042
	20	16.80	11.30	0.9952	0.049
50	10	22.60	10.80	0.9958	0.039
	15	22.50	10.80	0.9946	0.042
	20	16.50	11.20	0.9954	0.047
60	10	22.40	10.80	0.9959	0.039
	15	22.00	10.80	0.9951	0.043
	20	12.70	10.50	0.9972	0.046

Plasma treatment		K_1_ × 10^–3^ (h % ^−1^)	K_2_ × 10^–3^ (%^−1^)	R^2^	RMSE (%)^a^
Plasma power(W)	Exposure time (min)				
Digvijay					
Control	*–*	29.40	11.30	0.9911	0.046
40	10	24.50	11.10	0.9940	0.042
	15	23.60	11.10	0.9943	0.042
	20	15.70	11.10	0.9971	0.033
50	10	12.80	11.10	0.9947	0.068
	15	10.70	11.20	0.9993	0.016
	20	10.40	10.60	0.9962	0.057
60	10	8.40	11.60	0.9995	0.017
	15	7.30	11.20	0.9995	0.018
	20	6.60	11.00	0.9993	0.024
Rajas					
Control	–	32.30	10.70	0.9913	0.050
40	10	32.30	10.40	0.9853	0.064
	15	23.90	11.00	0.9949	0.038
	20	12.40	11.20	0.9982	0.041
50	10	13.10	11.50	0.9958	0.056
	15	13.00	11.30	0.9961	0.054
	20	9.70	11.50	0.9984	0.036
60	10	12.50	11.50	0.9966	0.049
	15	10.80	11.40	0.9978	0.044
	20	9.10	11.40	0.9990	0.029

^a^Root mean square error, %, $RMSE = 100* \sqrt {\frac{1}{n} \mathop \sum \limits_{1}^{n} [(M_{exp} - M_{pre} )/M_{exp} ]^{2} }$.

The water uptake fitted by the Peleg non-linear equation with coefficients shows that the water content of the seeds increased with the soaking time for both the plasma treated seeds and the untreated seeds. Peleg model was successfully fitted to correlate the water uptake of six chickpea cultivars. With increasing soaking time and cold plasma treatment, the R_2_ ranged from 0.9723 to 0.9999 and the RMSE (%) ranged from 0.010 to 0.098 for all the chickpea cultivars (Table 2) (Supplementary Table S3). Thus, the coefficient of determination (R^2^) and root mean square error percentage (RMSE) shown in Table 2 were more than 0.9 and less than 1%, respectively. Consequently, it is an indication that Peleg model was reliable enough to predict the moisture content of plasma-treated and control chickpeas^41^.

### Assessment of Peleg rate constant K_1_

K_1_ (Peleg rate constant) is a constant related to the rate of mass transfer, e.g. the lower the K_1_, the higher the initial rate of water uptake. K_1_ values were expressed as × 10^–3^ (h % − 1). K_1_ values for control samples of *Rajas* were 32.30 to 24.00 for all chickpea samples. K_1_ for plasma treated samples at 40, 50 and 60 watts for 10, 15 and 20 min respectively decreased from 24 to 9.80 for *Kripa*, 32.2 to 4.30 for *Virat*, 31.80 to 12.5 for *Vishal*, 29.3 to 12.7 for *Vijay*, 29.4 to 6.6 for *Digvijay* and 32.3 to 9.10 for *Rajas* with increase in plasma power and exposure time (Table 2) (Supplementary Table S3).

Peleg model K_1_ values of all plasma treated samples were lower than control (Fig. 3). Also, K_1_ values decreased progressively with increasing plasma power and exposure time for all six chickpea cultivars. This suggests that the surface wettability of the seeds was increased by the plasma treatment. Also, the treated seeds may have an eroded surface due to the plasma treatment resulting in increased initial water uptake rates^35^.

The sensitivity of K_1_ to plasma power and exposure time indicates the positive effect of cold plasma power and treatment exposure time on water absorption rate. Similar observations have been reported for the soaking studies with increasing temperature of the soaking water^10^. Researchers stated that water absorption in chickpea and bean varieties increased with increasing temperature of distilled water used for soaking and Peleg model constants were then examined as a function of temperature^41,45,46^. In the case of bean cultivars, their results showed a linear decrease in the coefficients K_1_ and K_2_^41,46^. For chickpeas, only the coefficient K_1_ decreased linearly, while K_2_ decreased partially^41,45^. Our results are consistent with theirs^41,45,46^.

In the experiment investigating the effect of maize kernel damage on water absorption rate, it was found that the water absorption rate of the most damaged maize kernels was significantly higher than that of the undamaged kernels^47^. Sensitivity to cold plasma treatment was more pronounced at 60 watts for 20 min, where K_1_ value was lowest. As determined by K_1_ values, the initial water uptake rate was highest at 60 watts for 20 min, consistent with Fig. 2. Thus, cold plasma power and treatment exposure time had a positive effect on the water absorption rate of all plasma treated chickpea samples of all cultivars. The plasma-induced change in the surface chemistry of the treated seeds may be the reason for this increased rate of water absorption, wettability or hydrophilicity. Often this is caused by introducing new oxygen-containing functional groups^44^. Plasma treatment also affects the surface morphology, mainly through its etching mechanisms, resulting in increased surface roughness^49^. As a result of the change in surface chemistry or etching of the seed surface, wettability was increased^44^. The latter then led to improved or accelerated water uptake^48^.

### Assessment of Peleg capacity constant K_2_

K_2_ (Peleg capacity constant) is a constant related to the maximum water uptake capacity, i.e. the lower K_2_, the higher the water uptake capacity. K_2_ values were expressed as × 10^–3^ (% − 1). K_2_ for control samples of all chickpea cultivars was found to be 10.40 to 12 for all chickpea cultivars (Table 2) (Supplementary Table S3). K_2_ for plasma treated chickpea cultivars at 40, 50 and 60 watts for 10, 15 and 20 min respectively ranged from 9.4 to 12 for all chickpea cultivars (Table 2) (Supplementary Table S3). Peleg's model K_2_ values of all plasma-treated and control samples were not different for all chickpea cultivars (Fig. 3). K_2_ values did not show a linear decrease with increase in plasma power and treatment time. It shows that plasma power and treatment time only improved the rate of water uptake and not the capacity to absorb water.

In addition, no effect of plasma power and treatment time on K_2_ was observed for any of the six chickpea cultivars. The effect of soaking temperature on water absorption was investigated in a few experiments. Researchers reported mixed effects of soaking water temperature on moisture absorption capacity K_2_. It depends on material and soluble solids loss. Shafaei et al. (2016) reported that the water absorption of chickpea and bean cultivars increased with the increase of soaking water temperature. They stated that the obtained Peleg model constants were investigated in relation to temperature. In the case of bean varieties, their results showed a linear decrease in the coefficients K_1_ and K_2_. For chickpeas, only the coefficient of K_1_ decreased linearly, while K_2_ decreased partially^41^. Many scientists have found that K_2_ is not affected by temperature^10,13,14,16,22,50,51^. At the same time, Turhan et al. (2002) reported an increase in K_2_ with increase in temperature^7^. In this study, neither plasma power nor exposure time showed any effect on K_2_.

### Comparison of K_1_ with K_2_

**Figure 4:**
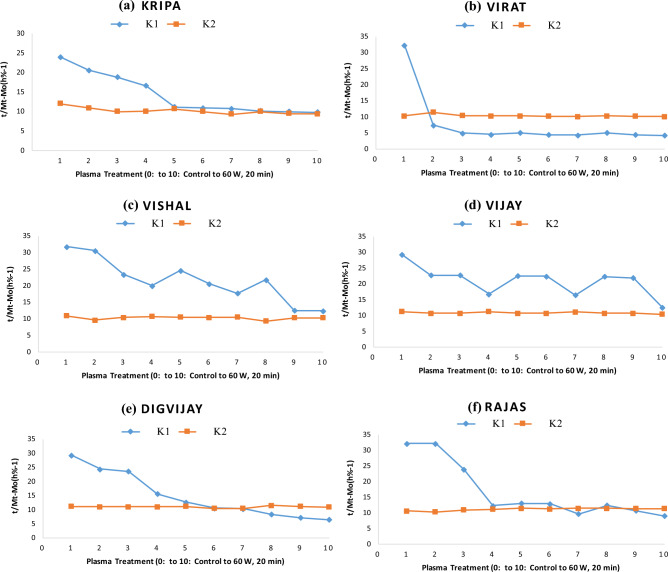
Comparison of K_1_ and K_2_ of plasma untreated and cold plasma treated six chickpea cultivars during soaking in distilled water.

To study the comparison of the Peleg rate constant (K_1_) with the Peleg capacity constant (K_2_), the plot of K_1_ and K_2_ of the control and the samples treated from 40 watts 10 min to 60 watts 20 min was prepared (Fig. 4). K_1_ values were expressed as × 10^–3^ (h % − 1). K_1_ decreased within the range of 32.3 to 4.30 for all chickpea cultivars (Table 2) in plasma-treated samples at 40, 50 and 60 W for 10, 15 and 20 min, respectively (Supplementary Table S3).

For the chickpea cultivars treated with plasma at 40, 50 and 60 watts for 10, 15 and 20 min respectively, K_2_ values range d from 9.4 to 12 for all the chickpea cultivars (Table 2). K_2_ values for all plasma-treated and control samples were not different or showed an increasing or decreasing trend for all chickpeas (Table 2) (Supplementary Table S3).

## Conclusions

Peleg model has been successfully fitted for the correlation of water absorption of plasma treated six chickpea cultivars. Peleg model satisfactorily predicted water absorption behaviour of cold plasma-treated chickpeas for different cultivars. At the beginning of soaking, when chickpeas were immersed in water, the moisture content increased rapidly. The rate of water absorption slowed down and the moisture content reached the saturation point in the course of time. Peleg rate constant K_1_ decreased with increasing plasma power and exposure time for all chickpea varieties. Peleg moisture capacity constant K_2_ was not affected by plasma power and exposure time for all chickpea varieties. It indicates that plasma treatment could cause beneficial changes in chickpea seeds, such as surface etching, which is responsible for the constant decrease in K_1_ with increasing plasma power and exposure time for all chickpea varieties. Thus, the K_1_ was lowest at 60W 20 min treatment for all chickpea varieties and found highest in control samples.

## Supplementary Information

Supplementary Information 1.

Supplementary Information 2.

Supplementary Information 3.

## Data Availability

The authors declare that data supporting the findings of this study are available within the paper and its supplementary Information files. Source data are provided in this paper.

## Abbreviations

K_1_: Peleg rate constant, h % d.b.^−^^1^
K_2_: Peleg capacity constant, % d.b.^−^^1^
M_t_: Moisture content at time t, % d.b.
M_0_: Initial moisture content, % d.b.
M_e_: Equilibrium moisture content, % d.b.
M_exp_: Experimental moisture content, % d.b.
M_pre_: Predicted moisture content, % d.b.
R^2^: Co-efficient of determination.
RMSE: Root Mean Square Error
W: Plasma power, watt
T: Time, hours
N: Number of observations
d.b.: Dry Basis
