# Supplementary material for: A peleg modeling of water absorption in cold plasma-treated Chickpea (Cicer arietinum L.) cultivars
Source: Sci Rep. 2023 May 15;13:7857. doi: 10.1038/s41598-023-33802-y (PMC10185522; doi:10.1038/s41598-023-33802-y)
Supplement: Supplementary file 1 — Supplementary Information 1. [file 41598_2023_33802_MOESM1_ESM.pdf]

Supplementary Table S1 Increase in weight (g) of plasma treated chickpea cultivars during soaking in distilled water

Plasma Treatment		Increase in Weight (g)									
Plasma Power (W)	Exposure Time (min.)	0 hr.	1 hr.	2 hrs.	3 hrs.	4 hrs.	5 hrs.	6 hrs.	7 hrs.	8 hrs.	9 hrs.
Kripa											
Control		8	9.22	10.9	12.07	13	13.55	14.02	15.04	15.05	15.05
		8	9.67	11.04	13.06	13.98	13.95	14.89	15.1	15.23	15.23
		8	9.33	10.72	12.03	12.73	13	14.2	14.73	14.94	14.94
		**8**	**9.406667**	**10.88667**	**12.38667**	**13.23667**	**13.5**	**14.37**	**14.95667**	**15.07333**	**15.07333**
40	10	8	9.45	11.39	13.08	14.19	14.87	15.58	15.86	16	16
		8	10.02	12.02	13.29	14.24	14.84	15.45	15.64	15.92	15.92
		8	10	12.07	13.19	14.14	14.81	15.49	15.69	16.07	16.07
		**8**	**9.823333**	**11.82667**	**13.18667**	**14.19**	**14.84**	**15.50667**	**15.73**	**15.99667**	**15.99667**
	15	8	11.32	12.75	13.6	14.71	15.29	15.92	16.7	16.74	16.74
		8	11.36	12.5	13.56	14.67	15.44	15.86	16.72	16.64	16.64
		8	10.99	12.7	13.52	14.64	15.4	15.84	16.56	16.67	16.67
		**8**	**11.22333**	**12.65**	**13.56**	**14.67333**	**15.37667**	**15.87333**	**16.66**	**16.68333**	**16.68333**
	20	8	10.93	12.9	14.03	14.91	15.63	16.23	16.73	16.77	16.77
		8	11.28	12.84	13.82	14.83	15.53	15.88	16.59	16.62	16.62
		8	11.48	12.98	13.77	14.7	15.56	15.96	16.84	16.87	16.87
		**8**	**11.23**	**12.90667**	**13.87333**	**14.81333**	**15.57333**	**16.02333**	**16.72**	**16.75333**	**16.75333**
Plasma Treatment		Increase in Weight (g)									
Plasma Power (W)	Exposure Time (min.)	0 hr.	1 hr.	2 hrs.	3 hrs.	4 hrs.	5 hrs.	6 hrs.	7 hrs.	8 hrs.	9 hrs.
Kripa											
Control		8	9.22	10.9	12.07	13	13.55	14.02	15.04	15.05	15.05
		8	9.67	11.04	13.06	13.98	13.95	14.89	15.1	15.23	15.23
		8	9.33	10.72	12.03	12.73	13	14.2	14.73	14.94	14.94
			**9.406667**	**10.88667**	**12.38667**	**13.23667**	**13.5**	**14.37**	**14.95667**	**15.07333**	**15.07333**
50	10	8	11.53	13.14	14.21	15.93	15.74	15.945	15.75	15.87	15.87
		8	11.881	13.04	14.11	15.83	15.64	15.845	15.809	16	16
		8	11.8	13.19	13.19	14.3	15.78	15.891	17.05	17.15	17.15
		**8**	**11.737**	**13.12333**	**13.83667**	**15.35333**	**15.72**	**15.89367**	**16.203**	**16.33933**	**16.33933**
	15	8	11.99	13.37	14.66	15.99	16.384	16.78	17.45	17.75	17.75
		8	11.88	13.471	14.751	15.99	16.23	16.99	16.998	17.15	17.15
		8	11.79	13.578	14.875	15.81	16.27	16.89	17.034	17.191	17.191
		**8**	**11.88667**	**13.473**	**14.762**	**15.93**	**16.29467**	**16.88667**	**17.16067**	**17.36367**	**17.36367**
	20	8	11.99	13.94	15.2	16.55	17.484	17.981	18.1	18.201	18.201
		8	11.951	13.941	15.25	16.649	17.335	17.99	18.15	18.29	18.29
		8	11.939	13.989	15.29	15.71	16.87	17.999	17.87	18.18	18.18
		**8**	**11.96**	**13.95667**	**15.24667**	**16.303**	**17.22967**	**17.99**	**18.04**	**18.22367**	**18.22367**

Plasma Treatment		Increase in Weight (g)									
Plasma Power (W)	Exposure Time (min.)	0 hr.	1 hr.	2 hrs.	3 hrs.	4 hrs.	5 hrs.	6 hrs.	7 hrs.	8 hrs.	9 hrs.
Kripa											
Control		8	9.22	10.9	12.07	13	13.55	14.02	15.04	15.05	15.05
		8	9.67	11.04	13.06	13.98	13.95	14.89	15.1	15.23	15.23
		8	9.33	10.72	12.03	12.73	13	14.2	14.73	14.94	14.94
		**8**	**9.406667**	**10.88667**	**12.38667**	**13.23667**	**13.5**	**14.37**	**14.95667**	**15.07333**	**15.07333**
60	10	8	11.9	13.16	14.25	15.61	15.49	15.681	16.25	16.26	16.26
		8	11.991	13.06	13.999	15.175	15.559	15.71	16.2	16.22	16.22
		8	11.39	13.21	13.768	15.285	16.765	16.998	16.48	16.51	16.51
		**8**	**11.76033**	**13.14333**	**14.00567**	**15.35667**	**15.938**	**16.12967**	**16.31**	**16.33**	**16.33**
	15	8	12.05	14.05	15.3	16.28	17.35	17.9	18	18.05	18.05
		8	11.99	14.1	15.32	16.3	17.39	17.993	18.03	18.09	18.09
		8	12	13.99	15.14	16.28	17.31	17.81	18.06	18.11	18.11
		**8**	**12.01333**	**14.04667**	**15.25333**	**16.28667**	**17.35**	**17.901**	**18.03**	**18.08333**	**18.08333**
	20	8	12.09	14.11	15.29	16.59	17.52	18.1	18.19	18.23	18.23
		8	12.06	14.1	15.32	16.679	17.375	18	18.15	18.1	18.1
		8	12.07	14.11	15.29	15.71	17.35	17.999	17.92	18.06	18.06
		**8**	**12.07333**	**14.10667**	**15.3**	**16.32633**	**17.415**	**18.033**	**18.08667**	**18.13**	**18.13**

Plasma Treatment		Increase in Weight (g)									
Plasma Power (W)	Exposure Time (min.)	0 hr.	1 hr.	2 hrs.	3 hrs.	4 hrs.	5 hrs.	6 hrs.	7 hrs.	8 hrs.	9 hrs.
Virat											
Control		8	9.09	10.8	12.05	13.2	14.05	14.4	14.99	15.08	15.08
		8	9.08	11.02	12.19	13.89	14.58	15.1	15.85	15.99	15.99
		8	9.2	10.92	12	13.04	13.9	14.09	15.02	15.15	15.15
		**8**	**9.123333**	**10.91333**	**12.11333**	**13.37667**	**14.17667**	**14.53**	**15.28667**	**15.40667**	**15.40667**
40	10	8	11.07	13.29	14.47	15.21	15.31	15.37	16.07	16.13	16.13
		8	11.35	13.05	15.3	15.52	16.05	16.16	16.34	16.48	16.48
		8	11.47	13.46	15.02	15.44	16.07	16.18	16.26	16.49	16.49
		**8**	**11.29667**	**13.26667**	**14.93**	**15.39**	**15.81**	**15.90333**	**16.22333**	**16.36667**	**16.36667**
	15	8	12.22	14.75	16.01	16.33	16.43	16.49	16.77	17.22	17.22
		8	12.74	14.85	16.34	16.64	16.76	16.81	16.99	17.51	17.51
		8	12.5	14.9	16.22	16.42	16.55	16.7	16.94	17.28	17.28
		**8**	**12.48667**	**14.83333**	**16.19**	**16.46333**	**16.58**	**16.66667**	**16.9**	**17.33667**	**17.33667**
	20	8	12.12	14.63	16.07	16.35	16.43	16.5	16.9	17.05	17.05
		8	12.76	15.09	16.31	16.7	16.71	16.72	16.88	17.43	17.43
		8	12.84	15.11	16.42	16.78	16.84	17.04	17.35	17.68	17.68
		**8**	**12.57333**	**14.94333**	**16.26667**	**16.61**	**16.66**	**16.75333**	**17.04333**	**17.38667**	**17.38667**

Plasma Treatment		Increase in Weight (g)									
Plasma Power (W)	Exposure Time (min.)	0 hr.	1 hr.	2 hrs.	3 hrs.	4 hrs.	5 hrs.	6 hrs.	7 hrs.	8 hrs.	9 hrs.
Virat											
Control		8	9.09	10.8	12.05	13.2	14.05	14.4	14.99	15.08	15.08
		8	9.08	11.02	12.19	13.89	14.58	15.1	15.85	15.99	15.99
		8	9.2	10.92	12	13.04	13.9	14.09	15.02	15.15	15.15
		**8**	**9.123333**	**10.91333**	**12.11333**	**13.37667**	**14.17667**	**14.53**	**15.28667**	**15.40667**	**15.40667**
50	10	8	12.36	14.9	16.07	16.34	16.41	16.98	17.31	17.36	17.36
		8	12.54	15.14	16.25	16.58	16.6	16.72	16.86	17.15	17.15
		8	12.43	15.05	16.12	16.42	16.51	16.75	17.21	17.46	17.46
		**8**	**12.44333**	**15.03**	**16.14667**	**16.44667**	**16.50667**	**16.81667**	**17.12667**	**17.32333**	**17.32333**
	15	8	12.52	14.64	15.04	16.051	16.66	16.82	16.94	17.24	17.24
		8	12.79	15.39	16.45	16.89	17.03	17.14	17.23	17.55	17.55
		8	12.81	15.4	16.46	16.9	17.04	17.16	17.24	17.31	17.31
		**8**	**12.70667**	**15.14333**	**15.98333**	**16.61367**	**16.91**	**17.04**	**17.13667**	**17.36667**	**17.36667**
	20	8	12.62	14.74	15.14	16.151	16.76	16.92	17.04	17.34	17.34
		8	12.89	15.49	16.55	16.99	17.13	17.24	17.33	17.65	17.65
		8	12.91	15.5	16.56	17	17.14	17.26	17.34	17.41	17.41
		**8**	**12.80667**	**15.24333**	**16.08333**	**16.71367**	**17.01**	**17.14**	**17.23667**	**17.46667**	**17.46667**

Plasma Treatment					Increase in Weight (g)						
Plasma Power (W)	Exposure Time (min.)	0 hr.	1 hr.	2 hrs.	3 hrs.	4 hrs.	5 hrs.	6 hrs.	7 hrs.	8 hrs.	9 hrs.
Virat											
Control		8	9.09	10.8	12.05	13.2	14.05	14.4	14.99	15.08	15.08
		8	9.08	11.02	12.19	13.89	14.58	15.1	15.85	15.99	15.99
		8	9.2	10.92	12	13.04	13.9	14.09	15.02	15.15	15.15
		**8**	**9.123333**	**10.91333**	**12.11333**	**13.37667**	**14.17667**	**14.53**	**15.28667**	**15.40667**	**15.40667**
60	10	8	12.38	14.92	16.09	16.36	16.43	16.99	17.33	17.38	17.38
		8	12.57	15.17	16.28	16.61	16.63	16.75	16.88	17.18	17.18
		8	12.45	15.08	16.14	16.44	16.54	16.79	17.24	17.49	17.49
		**8**	**12.46667**	**15.05667**	**16.17**	**16.47**	**16.53333**	**16.84333**	**17.15**	**17.35**	**17.35**
	15	8	12.54	14.67	15.07	16.055	16.68	16.84	16.96	17.28	17.28
		8	12.85	15.41	16.48	16.93	17.07	17.18	17.26	17.57	17.57
		8	12.82	15.43	16.49	16.92	17.06	17.19	17.27	17.34	17.34
		8	**12.73667**	**15.17**	**16.01333**	**16.635**	**16.93667**	**17.07**	**17.16333**	**17.39667**	**17.39667**
	20	8	12.65	14.76	15.17	16.159	16.78	16.94	17.07	17.37	17.37
		8	12.91	15.51	16.57	17.01	17.15	17.26	17.35	17.67	17.67
		8	12.94	15.53	16.59	17.12	17.16	17.29	17.37	17.43	17.43
		**8**	**12.83333**	**15.26667**	**16.11**	**16.763**	**17.03**	**17.16333**	**17.26333**	**17.49**	**17.49**

Plasma Treatment		Increase in Weight (g)									
Plasma Power (W)	Exposure Time (min.)	0 hr.	1 hr.	2 hrs.	3 hrs.	4 hrs.	5 hrs.	6 hrs.	7 hrs.	8 hrs.	9 hrs.
Vishal											
Control		8	9.1	10	11.9	13	13.45	14.02	15.04	15.05	15.05
		8	9.1	10	12.7	13.88	13.95	14.89	15	15.13	15.13
		8	8.3	10.32	11.9	12.73	13	14.1	14.73	14.94	14.94
		**8**	**8.833333**	**10.10667**	**12.16667**	**13.20333**	**13.46667**	**14.33667**	**14.92333**	**15.04**	**15.04**
40	10	8	9.14	10.48	12.09	13.2	14.88	15.62	15.79	16.09	16.09
		8	8.21	9.42	12.3	14.2	14.85	15.39	15.56	15.83	15.83
		8	9.16	10.5	12.2	14.1	14.82	15.28	15.58	15.98	15.98
		**8**	**8.836667**	**10.13333**	**12.19667**	**13.83333**	**14.85**	**15.43**	**15.64333**	**15.96667**	**15.96667**
	15	8	9.07	10.15	12.55	14.06	15.52	15.64	15.89	16.12	16.12
		8	9.35	10.16	13.17	14.41	14.72	15.41	15.8	15.86	15.86
		8	9.07	10.17	12.58	14.09	14.7	15.3	15.89	16.01	16.01
		**8**	**9.163333**	**10.16**	**12.76667**	**14.18667**	**14.98**	**15.45**	**15.86**	**15.99667**	**15.99667**
	20	8	9.21	10.15	13.09	14.6	15.45	15.69	15.72	15.88	15.88
		8	9.28	10.59	12.52	14.32	15.16	15.71	15.95	16.07	16.07
		8	9.28	10.17	13.098	14.62	15.35	15.8	16	16.1	16.1
		**8**	**9.256667**	**10.30333**	**12.90267**	**14.51333**	**15.32**	**15.73333**	**15.89**	**16.01667**	**16.01667**

Plasma Treatment					Increase in Weight (g)						
Plasma Power (W)	Exposure Time (min.)	0 hr.	1 hr.	2 hrs.	3 hrs.	4 hrs.	5 hrs.	6 hrs.	7 hrs.	8 hrs.	9 hrs.
Vishal											
Control		8	9.1	10	11.9	13	13.45	14.02	15.04	15.05	15.05
		8	9.1	10	12.7	13.88	13.95	14.89	15	15.13	15.13
		8	8.3	10.32	11.39	12.73	13	14.1	14.73	14.94	14.94
		**8**	**8.833333**	**10.10667**	**11.99667**	**13.20333**	**13.46667**	**14.33667**	**14.92333**	**15.04**	**15.04**
50	10	8	9.16	10.48	13.13	14.44	15.5	15.85	15.97	16.1	16.1
		8	9	9.46	12.52	14	15	15.86	15.95	15.93	15.93
		8	9.16	10.5	13.098	14	15.1	15.8	15.91	16.08	16.08
		**8**	**9.106667**	**10.14667**	**12.916**	**14.14667**	**15.2**	**15.83667**	**15.94333**	**16.03667**	**16.03667**
	15	8	9.68	11.05	13.24	14.48	15.39	15.93	16.1	16.3	16.3
		8	9.18	11.07	13.44	14.04	15.51	15.95	16.1	16.36	16.36
		8	9.68	11	13.12	14.22	15.61	15.93	16.12	16.3	16.3
		**8**	**9.513333**	**11.04**	**13.26667**	**14.24667**	**15.50333**	**15.93667**	**16.10667**	**16.32**	**16.32**
	20	8	9.73	11.15	13.52	14.75	15.72	16.21	16.35	16.48	16.48
		8	9.63	11.21	13.57	14.66	15.43	16.23	16.37	16.46	16.46
		8	9.75	11.25	13.52	14.67	15.64	16.33	16.39	16.49	16.49
		**8**	**9.703333**	**11.20333**	**13.53667**	**14.69333**	**15.59667**	**16.25667**	**16.37**	**16.47667**	**16.47667**

Plasma Treatment											
Plasma Power (W)	Exposure Time (min.)	0 hr.	1 hr.	2 hrs.	3 hrs.	4 hrs.	5 hrs.	6 hrs.	7 hrs.	8 hrs.	9 hrs.
Vishal											
Control		8	9.1	10	11.9	13	13.45	14.02	15.04	15.05	15.05
		8	9.1	10	12.7	13.88	13.95	14.89	15	15.13	15.13
		8	8.3	10.32	11.9	12.73	13	14.1	14.73	14.94	14.94
		**8**	**8.833333**	**10.10667**	**12.16667**	**13.20333**	**13.46667**	**14.33667**	**14.92333**	**15.04**	**15.04**
60	10	8	9.94	11.08	13.29	14.65	15.62	16.54	16.77	16.88	16.88
		8	9.04	10.84	12.8	14.32	15.37	15.96	16.35	16.51	16.51
		8	9.06	11.22	12.93	14.62	15.64	16.53	16.79	16.99	16.99
		**8**	**9.346667**	**11.04667**	**13.00667**	**14.53**	**15.54333**	**16.34333**	**16.63667**	**16.79333**	**16.79333**
	15	8	9.26	10.86	13.76	15.11	15.93	16.45	16.71	16.89	16.89
		8	9.8	10.6	13.14	14.59	15.81	16.49	16.92	17.21	17.21
		8	9.28	10.89	13.78	15.14	15.95	16.48	16.84	16.895	16.895
		**8**	**9.446667**	**10.78333**	**13.56**	**14.94667**	**15.89667**	**16.47333**	**16.82333**	**16.99833**	**16.99833**
	20	8	9.46	11.74	13.76	15.16	15.93	16.65	16.8	16.89	16.89
		8	10.03	11.52	13.19	14.59	15.85	16.69	16.92	17.2	17.2
		8	10.01	11.64	13.79	15.14	15.95	16.58	16.77	16.895	16.895
		**8**	**9.833333**	**11.63333**	**13.58**	**14.96333**	**15.91**	**16.64**	**16.83**	**16.995**	**16.995**

Plasma Treatment				Increase in Weight (g)							
Plasma Power (W)	Exposure Time (min.)	0 hr.	1 hr.	2 hrs.	3 hrs.	4 hrs.	5 hrs.	6 hrs.	7 hrs.	8 hrs.	9 hrs.
Vijay											
Control		8	9.12	10.9	12.07	13.19	13.45	14.12	15.04	15.15	15.15
		8	9.77	11.06	13.06	13.88	14.05	14.89	15.09	15.13	15.13
		8	9.38	10.77	12.08	12.78	13.04	14.15	14.78	14.99	14.99
		**8**	**9.423333**	**10.91**	**12.40333**	**13.28333**	**13.51333**	**14.38667**	**14.97**	**15.09**	**15.09**
40	10	8	9.85	11	13.21	14.36	14.97	15.54	15.71	16.04	16.04
		8	8.96	10.76	12.72	13.92	14.62	15.31	15.48	15.78	15.78
		8	8.98	11.14	12.9	13.92	14.6	15.2	15.5	15.93	15.93
		**8**	**9.263333**	**10.96667**	**12.94333**	**14.06667**	**14.73**	**15.35**	**15.56333**	**15.91667**	**15.91667**
	15	8	9.36	10.94	12.99	13.92	14.78	15.49	15.76	15.95	15.95
		8	9.93	11.93	12.93	13.97	14.75	15.36	15.55	15.78	15.78
		8	9.96	11.98	12.92	13.96	14.72	15.4	15.6	15.93	15.93
		**8**	**9.75**	**11.61667**	**12.94667**	**13.95**	**14.75**	**15.41667**	**15.63667**	**15.88667**	**15.88667**
	20	8	9.63	11.64	13.62	14.65	15.35	15.8	16.07	16.19	16.19
		8	9.53	11.42	13.27	14.46	15.06	15.61	15.81	15.97	15.97
		8	9.5	11.54	13.42	14.5	15.25	15.7	15.9	15.91	15.91
		**8**	**9.553333**	**11.53333**	**13.43667**	**14.53667**	**15.22**	**15.70333**	**15.92667**	**16.02333**	**16.02333**

Plasma Treatment			Increase in Weight (g)								
Plasma Power (W)	Exposure Time (min.)	0 hr.	1 hr.	2 hrs.	3 hrs.	4 hrs.	5 hrs.	6 hrs.	7 hrs.	8 hrs.	9 hrs.
Vijay											
Control		8	9.12	10.9	12.07	13.19	13.45	14.12	15.04	15.15	15.15
		8	9.77	11.06	13.06	13.88	14.05	14.89	15.09	15.13	15.13
		8	9.38	10.77	12.08	12.78	13.04	14.15	14.78	14.99	14.99
		**8**	**9.423333**	**10.91**	**12.40333**	**13.28333**	**13.51333**	**14.38667**	**14.97**	**15.09**	**15.09**
50	10	8	9.88	11.03	13.24	14.39	15	15.57	15.74	16.07	16.07
		8	8.99	10.79	12.75	13.95	14.65	15.34	15.51	15.81	15.81
		8	9.01	11.17	12.93	13.95	14.63	15.23	15.53	15.96	15.96
		**8**	**9.293333**	**10.99667**	**12.97333**	**14.09667**	**14.76**	**15.38**	**15.59333**	**15.94667**	**15.94667**
	15	8	9.41	10.99	13.04	13.97	14.83	15.54	15.81	16	16
		8	9.98	11.98	12.98	14.02	14.8	15.41	15.6	15.83	15.83
		8	10.01	12.03	12.97	14.01	14.77	15.45	15.65	15.98	15.98
		**8**	**9.8**	**11.66667**	**12.99667**	**14**	**14.8**	**15.46667**	**15.68667**	**15.93667**	**15.93667**
	20	8	9.68	11.69	13.67	14.7	15.4	15.85	16.12	16.24	16.24
		8	9.58	11.47	13.32	14.51	15.11	15.66	15.86	16.02	16.02
		8	9.55	11.59	13.47	14.55	15.3	15.75	15.95	15.96	15.96
		**8**	**9.603333**	**11.58333**	**13.48667**	**14.58667**	**15.27**	**15.75333**	**15.97667**	**16.07333**	**16.07333**

Plasma Treatment			Increase in Weight (g)								
Plasma Power (W)	Exposure Time (min.)	0 hr.	1 hr.	2 hrs.	3 hrs.	4 hrs.	5 hrs.	6 hrs.	7 hrs.	8 hrs.	9 hrs.
Vijay											
Control		8	9.12	10.9	12.07	13.19	13.45	14.12	15.04	15.15	15.15
		8	9.77	11.06	13.06	13.88	14.05	14.89	15.09	15.13	15.13
		8	9.38	10.77	12.08	12.78	13.04	14.15	14.78	14.99	14.99
		**8**	**9.423333**	**10.91**	**12.40333**	**13.28333**	**13.51333**	**14.38667**	**14.97**	**15.09**	**15.09**
60	10	8	9.9	11.5	13.26	14.41	15.02	15.59	15.76	16.09	16.09
		8	9.01	10.81	12.77	13.97	14.67	15.36	15.53	15.83	15.83
		8	9.3	11.19	12.95	13.97	14.65	15.25	15.55	15.98	15.98
		**8**	**9.403333**	**11.16667**	**12.99333**	**14.11667**	**14.78**	**15.4**	**15.61333**	**15.96667**	**15.96667**
	15	8	9.41	10.99	13.04	13.97	14.83	15.54	15.81	16.11	16.11
		8	9.98	11.98	12.98	14.2	14.8	15.41	15.6	15.83	15.83
		8	10.01	12.03	12.97	14.1	14.77	15.45	15.65	15.98	15.98
		**8**	**9.8**	**11.66667**	**12.99667**	**14.09**	**14.8**	**15.46667**	**15.68667**	**15.97333**	**15.97333**
	20	8	9.26	10.81	13.71	15.11	15.88	16.46	16.6	16.84	16.84
		8	9.75	10.6	13.14	14.54	15.8	16.44	16.77	17.2	17.2
		8	9.23	10.84	17.73	15.09	15.9	16.43	16.59	16.84	16.84
		**8**	**9.413333**	**10.75**	**14.86**	**14.91333**	**15.86**	**16.44333**	**16.65333**	**16.96**	**16.96**

Plasma Treatment			Increase in Weight (g)								
Plasma Power (W)	Exposure Time (min.)	0 hr.	1 hr.	2 hrs.	3 hrs.	4 hrs.	5 hrs.	6 hrs.	7 hrs.	8 hrs.	9 hrs.
Digvijay											
Control		8	9.08	10.86	12.03	13.15	13.41	14.08	15	15.11	15.11
		8	9.77	11.06	13.06	13.88	14.05	14.89	15.09	15.13	15.13
		8	9.38	10.77	12.08	12.78	13.04	14.15	14.78	14.99	14.99
		**8**	**9.41**	**10.89667**	**12.39**	**13.27**	**13.5**	**14.37333**	**14.95667**	**15.07667**	**15.07667**
40	10	8	9.78	11.06	12.53	13.35	13.81	14.88	15.27	15.51	15.51
		8	10.16	11.76	13.86	14.08	14.45	15.16	15.49	15.63	15.63
		8	9.68	11	12.48	13.3	13.74	14.85	15.2	15.48	15.48
		**8**	**9.873333**	**11.27333**	**12.95667**	**13.57667**	**14**	**14.96333**	**15.32**	**15.54**	**15.54**
	15	8	9.88	11.16	12.63	13.45	13.91	14.98	15.37	15.61	15.61
		8	10.26	11.86	13.96	14.18	14.55	15.26	15.59	15.73	15.73
		8	9.78	11.1	12.58	13.4	13.84	14.95	15.3	15.58	15.58
		**8**	**9.973333**	**11.37333**	**13.05667**	**13.67667**	**14.1**	**15.06333**	**15.42**	**15.64**	**15.64**
	20	8	10.756	12.69	13.98	14.12	14.71	15.58	15.72	15.87	15.87
		8	11.18	12.4	13.63	14.86	15.44	16.1	16.37	16.51	16.51
		8	10.6	12.58	13.9	14	14.7	15.49	15.71	15.85	15.85
		**8**	**10.84533**	**12.55667**	**13.83667**	**14.32667**	**14.95**	**15.72333**	**15.93333**	**16.07667**	**16.07667**

Plasma Treatment											
Plasma Power (W)	Exposure Time (min.)	0 hr.	1 hr.	2 hrs.	3 hrs.	4 hrs.	5 hrs.	6 hrs.	7 hrs.	8 hrs.	9 hrs.
Digvijay											
Control		8	9.08	10.86	12.03	13.15	13.41	14.08	15	15.11	15.11
		8	9.77	11.06	13.06	13.88	14.05	14.89	15.09	15.13	15.13
		8	9.38	10.77	12.08	12.78	13.04	14.15	14.78	14.99	14.99
		**8**	**9.41**	**10.89667**	**12.39**	**13.27**	**13.5**	**14.37333**	**14.95667**	**15.07667**	**15.07667**
50	10	8	11.1	12.82	13.93	15.3	15.77	16.07	16.16	16.46	16.46
		8	11.08	12.37	13.56	14.89	15.45	15.71	15.81	15.95	15.95
		8	11.2	12.52	13.6	15.41	15.75	16.02	16.15	16.39	16.39
		**8**	**11.12667**	**12.57**	**13.69667**	**15.2**	**15.65667**	**15.93333**	**16.04**	**16.26667**	**16.26667**
	15	8	10.7	12.97	15.3	15.37	15.84	16.03	16.1	16.56	16.56
		8	10.6	12.74	13.95	15.29	15.78	16.07	16.19	16.69	16.69
		8	10.55	12.85	14.5	15.35	15.81	16.05	16.15	16.6	16.6
		**8**	**10.61667**	**12.85333**	**14.58333**	**15.33667**	**15.81**	**16.05**	**16.14667**	**16.61667**	**16.61667**
	20	8	11.99	13.07	14.27	15.6	16.1	16.3	16.45	16.54	16.54
		8	11.81	13.01	14.1	15.51	16.11	16.41	16.6	16.74	16.74
		8	11.91	13.05	14.15	15.45	16.12	16.39	16.6	16.6	16.6
		**8**	**11.90333**	**13.04333**	**14.17333**	**15.52**	**16.11**	**16.36667**	**16.55**	**16.62667**	**16.62667**

Plasma Treatment		Increase in Weight (g)									
Plasma Power (W)	Exposure Time (min.)	0 hr.	1 hr.	2 hrs.	3 hrs.	4 hrs.	5 hrs.	6 hrs.	7 hrs.	8 hrs.	9 hrs.
Digvijay											
Control		8	9.08	10.86	12.03	13.15	13.41	14.08	15	15.11	15.11
		8	9.77	11.06	13.06	13.88	14.05	14.89	15.09	15.13	15.13
		8	9.38	10.77	12.08	12.78	13.04	14.15	14.78	14.99	14.99
		**8**	**9.41**	**10.89667**	**12.39**	**13.27**	**13.5**	**14.37333**	**14.95667**	**15.07667**	**15.07667**
60	10	8	10.59	12.76	14.21	14.99	15.64	15.79	15.76	16.04	16.04
		8	11.5	13.33	14.93	15.64	15.62	16.08	16.27	16.41	16.41
		8	11.45	12.91	14.7	15.12	16.05	15.98	16.19	16.36	16.36
		**8**	**11.18**	**13**	**14.61333**	**15.25**	**15.77**	**15.95**	**16.07333**	**16.27**	**16.27**
	15	8	12.51	13.59	15.01	15.68	16.19	16.21	16.27	16.61	16.61
		8	11.35	13	14.55	15.19	15.77	15.89	16	16.34	16.34
		8	11.99	13.2	14.97	15.82	15.98	16.12	16.25	16.52	16.52
		**8**	**11.95**	**13.26333**	**14.84333**	**15.56333**	**15.98**	**16.07333**	**16.17333**	**16.49**	**16.49**
	20	8	12.07	13.46	15.2	15.99	16.23	16.33	16.45	16.61	16.61
		8	11.99	13.36	14.96	15.61	16.37	16.47	16.66	16.74	16.74
		8	12.03	13.38	14.94	15.68	16.25	16.39	16.6	16.6	16.6
		**8**	**12.03**	**13.4**	**15.03333**	**15.76**	**16.28333**	**16.39667**	**16.57**	**16.65**	**16.65**

Plasma Treatment		Increase in Weight (g)									
Plasma Power (W)	Exposure Time (min.)	0 hr.	1 hr.	2 hrs.	3 hrs.	4 hrs.	5 hrs.	6 hrs.	7 hrs.	8 hrs.	9 hrs.
Rajas											
Control		8	9.1	9.28	12	13.24	13.8	14.5	14.95	15.26	15.26
		8	9.4	10.6	12.12	13	14.28	14.4	14.99	15.23	15.23
		8	10.3	11.5	12.1	13.42	13.88	14.52	14.7	15.24	15.24
		**8**	**9.6**	**10.46**	**12.07333**	**13.22**	**13.98667**	**14.47333**	**14.88**	**15.24333**	**15.24333**
40	10	8	9.67	10.78	12.03	13.18	14.03	14.38	14.98	15.06	15.06
		8	9.66	11	12.17	13.87	14.56	15.08	15.82	15.98	15.98
		8	9.68	10.9	12	13.02	13.88	14.07	15	15.12	15.12
		**8**	**9.67**	**10.89333**	**12.09333**	**13.35667**	**14.15667**	**14.51**	**15.26667**	**15.38667**	**15.38667**
	15	8	9.57	11.4	12.83	13.78	14.33	14.8	15.13	15.28	15.28
		8	9.6	11.32	12.77	14.17	15.01	15.48	15.86	16.07	16.07
		8	9.58	11.34	13	13.42	14.21	14.87	15.54	15.59	15.59
		**8**	**9.583333**	**11.35333**	**12.79333**	**13.79**	**14.51667**	**15.05**	**15.51**	**15.64667**	**15.64667**
	20	8	11.33	12.58	13.72	14.52	15.13	15.54	15.82	15.91	15.91
		8	11.35	13.06	14.16	15.35	15.85	16.16	16.45	16.57	16.57
		8	11.34	12.787	13.88	14.74	15.24	15.72	15.88	15.98	15.98
		**8**	**11.34**	**12.809**	**13.92**	**14.87**	**15.40667**	**15.80667**	**16.05**	**16.15333**	**16.15333**

Plasma Treatment			Increase in Weight (g)								
Plasma Power (W)	Exposure Time (min.)	0 hr.	1 hr.	2 hrs.	3 hrs.	4 hrs.	5 hrs.	6 hrs.	7 hrs.	8 hrs.	9 hrs.
Rajas											
Control		8	9.1	9.28	12	13.24	13.8	14.5	14.95	15.26	15.26
		8	9.4	10.6	12.12	13	14.28	14.4	14.99	15.23	15.23
		8	10.3	11.5	12.1	13.42	13.88	14.52	14.7	15.24	15.24
		**8**	**9.6**	**10.46**	**12.07333**	**13.22**	**13.98667**	**14.47333**	**14.88**	**15.24333**	**15.24333**
50	10	8	10.05	12.88	13.72	15.08	15.61	15.86	15.93	16.2	16.2
		8	10.02	12.63	13.69	14.79	15.48	15.89	15.9	15.91	15.91
		8	10.04	12.7	13.72	14.82	15.5	15.72	15.92	16.12	16.12
		**8**	**10.03667**	**12.73667**	**13.71**	**14.89667**	**15.53**	**15.82333**	**15.91667**	**16.07667**	**16.07667**
	15	8	10.04	13.04	14.16	15.17	15.69	16.14	16.19	16.35	16.35
		8	10	12.65	13.59	14.89	15.48	15.69	15.79	16.05	16.05
		8	10.03	12.8	13.68	14.97	15.68	16.12	16.21	16.31	16.31
		**8**	**10.02333**	**12.83**	**13.81**	**15.01**	**15.61667**	**15.98333**	**16.06333**	**16.23667**	**16.23667**
	20	8	10.04	13.56	14.28	15.13	15.66	15.98	16.2	16.29	16.29
		8	10.06	13.27	14.13	15.14	15.68	16.12	16.04	16.15	16.15
		8	10.03	13.5	14.42	15.54	15.65	15.99	16.13	16.28	16.28
		**8**	**10.04333**	**13.44333**	**14.27667**	**15.27**	**15.66333**	**16.03**	**16.12333**	**16.24**	**16.24**

Plasma Treatment			Increase in Weight (g)								
Plasma Power (W)	Exposure Time (min.)	0 hr.	1 hr.	2 hrs.	3 hrs.	4 hrs.	5 hrs.	6 hrs.	7 hrs.	8 hrs.	9 hrs.
Rajas											
Control		8	9.1	9.28	12	13.24	13.8	14.5	14.95	15.26	15.26
		8	9.4	10.6	12.12	13	14.28	14.4	14.99	15.23	15.23
		8	10.3	11.5	12.1	13.42	13.88	14.52	14.7	15.24	15.24
		**8**	**9.6**	**10.46**	**12.07333**	**13.22**	**13.98667**	**14.47333**	**14.88**	**15.24333**	**15.24333**
60	10	8	10.05	13.04	14.02	15.13	15.61	15.86	15.97	16.26	16.26
		8	10.06	12.69	13.69	14.79	15.56	15.89	15.99	15.91	15.91
		8	10.04	12.8	13.77	14.82	15.5	15.77	15.95	16.12	16.12
		**8**	**10.05**	**12.84333**	**13.82667**	**14.91333**	**15.55667**	**15.84**	**15.97**	**16.09667**	**16.09667**
	15	8	10.05	12.73	14.1	15.27	15.84	16.07	16.14	16.33	16.33
		8	10	13.76	14.15	15.36	15.54	15.98	16.1	16.26	16.26
		8	10.04	12.98	14.12	15.28	15.62	15.99	16.11	16.29	16.29
		**8**	**10.03**	**13.15667**	**14.12333**	**15.30333**	**15.66667**	**16.01333**	**16.11667**	**16.29333**	**16.29333**
	20	8	10.13	13.96	14.8	15.63	15.66	15.98	16.26	16.38	16.38
		8	10.13	13.32	14.19	15.14	15.68	16.19	16.22	16.32	16.32
		8	10.15	13.5	14.42	15.61	15.71	15.99	16.13	16.33	16.33
		**8**	**10.13667**	**13.59333**	**14.47**	**15.46**	**15.68333**	**16.05333**	**16.20333**	**16.34333**	**16.34333**
