# Supplementary material for: A peleg modeling of water absorption in cold plasma-treated Chickpea (Cicer arietinum L.) cultivars
Source: Sci Rep. 2023 May 15;13:7857. doi: 10.1038/s41598-023-33802-y (PMC10185522; doi:10.1038/s41598-023-33802-y)
Supplement: Supplementary file 2 — Supplementary Information 2. [file 41598_2023_33802_MOESM2_ESM.pdf]

Supplementary Table S2 increase in Moisture (% db) of plasma treated chickpea cultivars during soaking in distilled water

Plasma Treatment		Moisture Content (% d.b.)									
Plasma Power (W)	Exposure Time (min.)	0 hr.	1 hr.	2 hrs.	3 hrs.	4 hrs.	5 hrs.	6 hrs.	7 hrs.	8 hrs.	9 hrs.
Kripa											
Control											
		8.4	17.58333	15.73352	13.77832	6.862217	1.989423	6.444444	4.082579	0.780031	0
		8.4	25.98333	41.71686	55.49518	62.3574	64.34682	70.79126	74.87384	75.65387	75.65387
40	10										
		8.4	22.79167	20.39362	11.49944	7.608696	4.580691	4.492363	1.440241	1.695274	0
		8.4	31.19167	51.58529	63.08472	70.69342	75.27411	79.76647	81.20671	82.90199	82.90199
	15	8.4	40.29167	12.71161	7.193676	8.210423	4.793276	3.230002	4.955901	0.140056	0
		8.4	48.69167	61.40328	68.59696	76.80738	81.60065	84.83066	89.78656	89.92661	89.92661
	20	8.4	40.375	14.93025	7.489669	6.775589	5.130513	2.889555	4.347826	0.199362	0
		8.4	48.775	63.70525	71.19492	77.9705	83.10102	85.99057	90.3384	90.53776	90.53776

Plasma Treatment		Moisture Content (% d.b.)									
Plasma Power (W)	Exposure Time (min.)	0 hr.	1 hr.	2 hrs.	3 hrs.	4 hrs.	5 hrs.	6 hrs.	7 hrs.	8 hrs.	9 hrs.
Kripa											
Control											
50	10	8.4	17.58333	15.73352	13.77832	6.862217	1.989423	6.444444	4.082579	0.780031	0
		8.4	25.98333	41.71686	55.49518	62.3574	64.34682	70.79126	74.87384	75.65387	75.65387
	15	8.4	46.7125	13.34549	5.435611	10.96121	2.388189	1.10475	1.946268	0.841408	0
		8.4	55.1125	68.45799	73.8936	84.85481	87.243	88.34775	90.29402	91.13543	91.13543
	20	8.4	48.58333	13.34549	9.567283	7.912207	2.289182	3.633091	1.622582	1.182938	0
		8.4	56.98333	70.32882	79.8961	87.80831	90.09749	93.73058	95.35316	96.5361	96.5361
		8.4	49.5	16.69454	9.242895	6.92829	5.684025	4.412931	0.277932	1.018108	0
		8.4	57.9	74.59454	83.83743	90.76572	96.44975	100.8627	101.1406	102.1587	102.1587

Plasma Treatment		Moisture Content (% d.b.)									
Plasma Power (W)	Exposure Time (min.)	0 hr.	1 hr.	2 hrs.	3 hrs.	4 hrs.	5 hrs.	6 hrs.	7 hrs.	8 hrs.	9 hrs.
Kripa											
Control											
		8.4	17.58333	15.73352	13.77832	6.862217	1.989423	6.444444	4.082579	0.780031	0
		8.4	25.98333	41.71686	55.49518	62.3574	64.34682	70.79126	74.87384	75.65387	75.65387
60	10										
		8.4	47.00417	11.75987	6.560994	9.646096	3.785544	1.202577	1.118023	0.122624	0
		8.4	55.40417	67.16404	73.72503	83.37113	87.15667	88.35925	89.47727	89.59989	89.59989
	15										
		8.4	50.16667	16.92564	8.590413	6.774476	6.528858	3.175793	0.72063	0.295803	0
		8.4	58.56667	75.4923	84.08272	90.85719	97.38605	100.5618	101.2825	101.5783	101.5783
	20										
		8.4	50.91667	16.84152	8.459357	6.708061	6.668164	3.548665	0.297603	0.239587	0
		8.4	59.31667	76.15819	84.61755	91.32561	97.99377	101.5424	101.84	102.0796	102.0796

Plasma Treatment

Plasma Power (W)	Exposure Time (min.)	Moisture Content (% d.b.)									
		0 hr.	1 hr.	2 hrs.	3 hrs.	4 hrs.	5 hrs.	6 hrs.	7 hrs.	8 hrs.	9 hrs.
Virat											
Control											
		8.2	14.04167	19.62002	10.99572	10.42928	5.980563	2.492358	5.207616	0.784998	0
		8.2	22.24167	41.86169	52.85741	63.28669	69.26725	71.75961	76.96723	77.75223	77.75223
40	10										
		8.2	41.20833	17.43877	12.53769	3.081045	2.729045	0.590344	2.012157	0.883501	0
		8.2	49.40833	66.84711	79.38479	82.46584	85.19488	85.78523	87.79738	88.68089	88.68089
	15										
		8.2	56.08333	18.79338	9.146067	1.688285	0.708645	0.522718	1.4	2.583826	0
		8.2	64.28333	83.07671	92.22278	93.91107	94.61971	95.14243	96.54243	99.12626	99.12626
	20										
		8.2	57.16667	18.84942	8.855677	2.110656	0.301023	0.560224	1.730999	2.014473	0
		8.2	65.36667	84.21608	93.07176	95.18242	95.48344	96.04366	97.77466	99.78914	99.78914

Plasma Treatment		Moisture Content (% d.b.)									
Plasma Power (W)	Exposure Time (min.)	0 hr.	1 hr.	2 hrs.	3 hrs.	4 hrs.	5 hrs.	6 hrs.	7 hrs.	8 hrs.	9 hrs.
Virat											
Control											
		8.2	14.04167	19.62002	10.99572	10.42928	5.980563	2.492358	5.207616	0.784998	0
		8.2	22.24167	41.86169	52.85741	63.28669	69.26725	71.75961	76.96723	77.75223	77.75223
50	10										
		8.2	55.54167	20.78757	7.429585	1.857969	0.364816	1.878029	1.843409	1.148307	0
		8.2	63.74167	84.52924	91.95882	93.81679	94.18161	96.05964	97.90304	99.05135	99.05135
	15										
		8.2	58.83333	19.17629	5.546995	3.943691	1.783672	0.768776	0.567293	1.342151	0
		8.2	67.03333	86.20962	91.75661	95.70031	97.48398	98.25275	98.82005	100.1622	100.1622
	20										
		8.2	60.08333	19.02655	5.510606	3.919171	1.773	0.764256	0.563983	1.334365	0
		8.2	68.28333	87.30988	92.82049	96.73966	98.51266	99.27692	99.8409	101.1753	101.1753

Plasma Treatment		Moisture Content (% d.b.)									
Plasma Power (W)	Exposure Time (min.)	0 hr.	1 hr.	2 hrs.	3 hrs.	4 hrs.	5 hrs.	6 hrs.	7 hrs.	8 hrs.	9 hrs.
Virat											
Control											
		8.2	14.04167	19.62002	10.99572	10.42928	5.980563	2.492358	5.207616	0.784998	0
		8.2	22.24167	41.86169	52.85741	63.28669	69.26725	71.75961	76.96723	77.75223	77.75223
60	10										
		8.2	55.83333	20.7754	7.394288	1.855288	0.384538	1.875	1.820701	1.166181	0
		8.2	64.03333	84.80873	92.20302	94.05831	94.44285	96.31785	98.13855	99.30473	99.30473
	15										
		8.2	59.20833	19.10495	5.559218	3.882182	1.813446	0.787247	0.546768	1.359487	0
		8.2	67.40833	86.51328	92.0725	95.95468	97.76812	98.55537	99.10214	100.4616	100.4616
	20										
		8.2	60.41667	18.96104	5.524017	4.053383	1.592794	0.782932	0.582637	1.312995	0
		8.2	68.61667	87.57771	93.10172	97.15511	98.7479	99.53083	100.1135	101.4265	101.4265

Plasma Treatment		Moisture Content (% d.b.)									
Plasma Power (W)	Exposure Time (min.)	0 hr.	1 hr.	2 hrs.	3 hrs.	4 hrs.	5 hrs.	6 hrs.	7 hrs.	8 hrs.	9 hrs.
Vishal											
Control											
		10	10.41667	14.41509	20.38259	8.520548	1.994446	6.460396	4.092072	0.781774	0
		10	20.41667	34.83176	55.21435	63.73489	65.72934	72.18974	76.28181	77.06358	77.06358
40	10										
		10	10.45833	14.67371	20.36184	13.41897	7.349398	3.905724	1.382588	2.066908	0
		10	20.45833	35.13204	55.49388	68.91285	76.26225	80.16797	81.55056	83.61747	83.61747
	15										
		10	14.54167	10.87668	25.65617	11.12272	5.592105	3.137517	2.653722	0.861707	0
		10	24.54167	35.41835	61.07452	72.19723	77.78934	80.92685	83.58058	84.44228	84.44228
	20										
		10	15.70833	11.30717	25.22808	12.48321	5.558107	2.697998	0.995763	0.797147	0
		10	25.70833	37.0155	62.24358	74.72679	80.2849	82.98289	83.97866	84.7758	84.7758

Plasma Treatment		Moisture Content (% d.b.)									
Plasma Power (W)	Exposure Time (min.)	0 hr.	1 hr.	2 hrs.	3 hrs.	4 hrs.	5 hrs.	6 hrs.	7 hrs.	8 hrs.	9 hrs.
Vishal											
Control											
		10	17.16667	15.8606	13.75077	6.880734	1.994446	6.460396	4.092072	0.781774	0
		10	27.16667	43.02726	56.77803	63.65877	65.65321	72.11361	76.20568	76.98745	76.98745
50	10										
		10	13.83333	11.4202	27.29304	9.528234	7.445806	4.188596	0.673542	0.585407	0
		10	23.83333	35.25354	62.54657	72.07481	79.52061	83.70921	84.38275	84.96816	84.96816
	15										
		10	18.91667	16.04765	20.16908	7.386935	8.820777	2.795098	1.066722	1.324503	0
		10	28.91667	44.96432	65.1334	72.52034	81.34111	84.13621	85.20293	86.52744	86.52744
	20										
		10	21.29167	15.45861	20.82713	8.544693	6.147913	4.231673	0.69715	0.651598	0
		10	31.29167	46.75027	67.57741	76.1221	82.27001	86.50169	87.19884	87.85043	87.85043

Plasma Treatment		Moisture Content (% d.b.)									
Plasma Power (W)	Exposure Time (min.)	0 hr.	1 hr.	2 hrs.	3 hrs.	4 hrs.	5 hrs.	6 hrs.	7 hrs.	8 hrs.	9 hrs.
Vishal											
Control											
		10	17.16667	15.8606	13.75077	6.880734	1.994446	6.460396	4.092072	0.781774	0
		10	27.16667	43.02726	56.77803	63.65877	65.65321	72.11361	76.20568	76.98745	76.98745
60	10										
		10	16.83333	18.1883	17.74291	11.71194	6.974077	5.146901	1.794819	0.941695	0
		10	26.83333	45.02164	62.76454	74.47649	81.45056	86.59746	88.39228	89.33398	89.33398
	15										
		10	18.08333	14.14961	38.11437	0.358102	6.355932	3.627595	2.124646	1.040222	0
		10	28.08333	42.23295	80.34732	80.70542	87.06135	90.68895	92.81359	93.85382	93.85382
	20										
		10	22.91667	18.30508	28.16619	0.357702	6.326576	4.588309	1.141827	0.980392	0
		10	32.91667	51.22175	79.38794	79.74564	86.07222	90.66053	91.80235	92.78275	92.78275

Plasma Treatment		Moisture Content (% d.b.)									
Plasma Power (W)	Exposure Time (min.)	0 hr.	1 hr.	2 hrs.	3 hrs.	4 hrs.	5 hrs.	6 hrs.	7 hrs.	8 hrs.	9 hrs.
Vijay											
Control		10.1	17.79167	15.77644	13.68775	7.094867	1.731493	6.462753	4.05468	0.801603	0
		10.1	27.89167	43.66811	57.35586	64.45072	66.18222	72.64497	76.69965	77.50125	77.50125
40	10										
		10.1	15.79167	18.38791	18.02432	8.678857	4.71564	4.209097	1.389794	2.270293	0
		10.1	25.89167	44.27958	62.30389	70.98275	75.69839	79.90749	81.29728	83.56757	83.56757
	15										
		10.1	21.875	19.1453	11.44907	7.749743	5.734767	4.519774	1.427027	1.598806	0
		10.1	31.975	51.1203	62.56937	70.31911	76.05388	80.57365	82.00068	83.59948	83.59948
	20										
		10.1	19.41667	20.72575	16.50289	8.186554	4.700757	3.175646	1.422203	0.606949	0
		10.1	29.51667	50.24242	66.74531	74.93186	79.63262	82.80826	84.23047	84.83742	84.83742

Plasma Treatment		Moisture Content (% d.b.)									
Plasma Power (W)	Exposure Time (min.)	0 hr.	1 hr.	2 hrs.	3 hrs.	4 hrs.	5 hrs.	6 hrs.	7 hrs.	8 hrs.	9 hrs.
Vijay											
Control											
50	10	10.1	17.79167	15.77644	13.68775	7.094867	1.731493	6.462753	4.05468	0.801603	0
		10.1	27.89167	43.66811	57.35586	64.45072	66.18222	72.64497	76.69965	77.50125	77.50125
	15	10.1	16.16667	18.32855	17.97514	8.658787	4.705604	4.200542	1.387083	2.265926	0
		10.1	26.26667	44.59522	62.57036	71.22915	75.93475	80.1353	81.52238	83.7883	83.7883
	20	10.1	22.5	19.04762	11.4	7.719928	5.714286	4.504505	1.422414	1.59371	0
		10.1	32.6	51.64762	63.04762	70.76755	76.48183	80.98634	82.40875	84.00246	84.00246
		10.1	20.04167	20.61784	16.43165	8.156204	4.684644	3.165248	1.417689	0.605049	0
		10.1	30.14167	50.75951	67.19116	75.34737	80.03201	83.19726	84.61495	85.22	85.22

Plasma Treatment		Moisture Content (% d.b.)									
Plasma Power (W)	Exposure Time (min.)	0 hr.	1 hr.	2 hrs.	3 hrs.	4 hrs.	5 hrs.	6 hrs.	7 hrs.	8 hrs.	9 hrs.
Vijay											
Control											
		10.1	17.79167	15.77644	13.68775	7.094867	1.731493	6.462753	4.05468	0.801603	0
		10.1	27.89167	43.66811	57.35586	64.45072	66.18222	72.64497	76.69965	77.50125	77.50125
60	10										
		10.1	17.54167	18.75222	16.35821	8.645459	4.698937	4.194858	1.385281	2.263023	0
		10.1	27.64167	46.39388	62.75209	71.39755	76.09649	80.29135	81.67663	83.93965	83.93965
	15										
		10.1	22.5	19.04762	11.4	8.412413	5.039035	4.504505	1.422414	1.827454	0
		10.1	32.6	51.64762	63.04762	71.46003	76.49907	81.00357	82.42599	84.25344	84.25344
	20										
		10.1	17.66667	14.19972	38.23256	0.358905	6.347787	3.678016	1.277113	1.841473	0
		10.1	27.76667	41.96638	80.19894	80.55785	86.90563	90.58365	91.86076	93.70224	93.70224

Plasma Treatment		Moisture Content (% d.b.)									
Plasma Power (W)	Exposure Time (min.)	0 hr.	1 hr.	2 hrs.	3 hrs.	4 hrs.	5 hrs.	6 hrs.	7 hrs.	8 hrs.	9 hrs.
Digvijay											
Control											
		9.3	17.625	15.7988	13.7045	7.102502	1.733233	6.469136	4.058442	0.802318	0
		9.3	26.925	42.7238	56.42829	63.53079	65.26403	71.73316	75.7916	76.59392	76.59392
40	10										
		9.3	23.41667	14.17961	14.93199	4.785181	3.118095	6.880952	2.383604	1.436031	0
		9.3	32.71667	46.89628	61.82827	66.61345	69.73154	76.6125	78.9961	80.43213	80.43213
	15										
		9.3	24.66667	14.03743	14.8007	4.748532	3.095296	6.832151	2.36778	1.426719	0
		9.3	33.96667	48.0041	62.8048	67.55334	70.64863	77.48078	79.84856	81.27528	81.27528
	20										
		9.3	35.56667	15.77944	10.19379	3.541315	4.350861	5.172798	1.335595	0.899582	0
		9.3	44.86667	60.64611	70.8399	74.38121	78.73208	83.90487	85.24047	86.14005	86.14005

Plasma Treatment		Moisture Content (% d.b.)									
Plasma Power (W)	Exposure Time (min.)	0 hr.	1 hr.	2 hrs.	3 hrs.	4 hrs.	5 hrs.	6 hrs.	7 hrs.	8 hrs.	9 hrs.
Digvijay											
Control											
		9.3	17.625	15.7988	13.7045	7.102502	1.733233	6.469136	4.058442	0.802318	0
		9.3	26.925	42.7238	56.42829	63.53079	65.26403	71.73316	75.7916	76.59392	76.59392
50	10										
		9.3	39.08333	12.97184	8.96314	10.97591	3.004386	1.767085	0.669456	1.413134	0
		9.3	48.38333	61.35517	70.31831	81.29422	84.29861	86.06569	86.73515	88.14828	88.14828
	15										
		9.3	32.70833	21.0675	13.45954	5.165714	3.086286	1.518027	0.602285	2.910818	0
		9.3	42.00833	63.07584	76.53538	81.7011	84.78738	86.30541	86.90769	89.81851	89.81851
	20										
		9.3	48.79167	9.577149	8.66343	9.501411	3.801546	1.593213	1.120163	0.463243	0
		9.3	58.09167	67.66882	76.33225	85.83366	89.6352	91.22842	92.34858	92.81182	92.81182

Plasma Treatment		Moisture Content (% d.b.)									
Plasma Power (W)	Exposure Time (min.)	0 hr.	1 hr.	2 hrs.	3 hrs.	4 hrs.	5 hrs.	6 hrs.	7 hrs.	8 hrs.	9 hrs.
Digvijay											
Control											
		9.3	17.625	15.7988	13.7045	7.102502	1.733233	6.469136	4.058442	0.802318	0
		9.3	26.925	42.7238	56.42829	63.53079	65.26403	71.73316	75.7916	76.59392	76.59392
60	10										
		9.3	39.75	16.27907	12.41026	4.356752	3.409836	1.141408	0.77325	1.223559	0
		9.3	49.05	65.32907	77.73933	82.09608	85.50591	86.64732	87.42057	88.64413	88.64413
	15										
		9.3	49.375	10.99024	11.91254	4.850662	2.677233	0.584063	0.622148	1.957955	0
		9.3	58.675	69.66524	81.57778	86.42844	89.10567	89.68974	90.31189	92.26984	92.26984
	20										
		9.3	50.375	11.3882	12.18905	4.833703	3.320643	0.696008	1.057125	0.4828	0
		9.3	59.675	71.0632	83.25225	88.08595	91.4066	92.1026	93.15973	93.64253	93.64253

Plasma Treatment		Moisture Content (% d.b.)									
Plasma Power (W)	Exposure Time (min.)	0 hr.	1 hr.	2 hrs.	3 hrs.	4 hrs.	5 hrs.	6 hrs.	7 hrs.	8 hrs.	9 hrs.
Rajas											
Control											
		9.1	20	8.958333	15.42384	9.497515	5.799294	3.479504	2.809765	2.441756	0
		9.1	29.1	38.05833	53.48217	62.97969	68.77898	72.25848	75.06825	77.51001	77.51001
40	10										
		9.1	20.875	12.65081	11.01591	10.44653	5.989518	2.495879	5.214794	0.786026	0
		9.1	29.975	42.62581	53.64172	64.08825	70.07777	72.57365	77.78844	78.57447	78.57447
	15										
		9.1	19.79167	18.46957	12.6835	7.790516	5.269519	3.673938	3.056478	0.881152	0
		9.1	28.89167	47.36123	60.04473	67.83525	73.10477	76.7787	79.83518	80.71633	80.71633
	20										
		9.1	41.75	12.95414	8.673589	6.824713	3.609056	2.596279	1.539435	0.643821	0
		9.1	50.85	63.80414	72.47773	79.30245	82.9115	85.50778	87.04722	87.69104	87.69104

Plasma Treatment		Moisture Content (% d.b.)									
Plasma Power (W)	Exposure Time (min.)	0 hr.	1 hr.	2 hrs.	3 hrs.	4 hrs.	5 hrs.	6 hrs.	7 hrs.	8 hrs.	9 hrs.
Rajas											
Control											
		9.1	20	8.958333	15.42384	9.497515	5.799294	3.479504	2.809765	2.441756	0
		9.1	29.1	38.05833	53.48217	62.97969	68.77898	72.25848	75.06825	77.51001	77.51001
50	10										
		9.1	25.45833	26.90136	7.641979	8.655483	4.25151	1.888817	0.589846	1.005236	0
		9.1	34.55833	61.4597	69.10167	77.75716	82.00867	83.89748	84.48733	85.49257	85.49257
	15										
		9.1	25.29167	28.00133	7.638348	8.689356	4.04175	2.347919	0.500521	1.079062	0
		9.1	34.39167	62.393	70.03134	78.7207	82.76245	85.11037	85.61089	86.68995	86.68995
	20										
		9.1	25.54167	33.8533	6.198859	6.95774	2.575857	2.340924	0.582242	0.723589	0
		9.1	34.64167	68.49497	74.69383	81.65157	84.22743	86.56835	87.15059	87.87418	87.87418

Plasma Treatment		Moisture Content (% d.b.)									
Plasma Power (W)	Exposure Time (min.)	0 hr.	1 hr.	2 hrs.	3 hrs.	4 hrs.	5 hrs.	6 hrs.	7 hrs.	8 hrs.	9 hrs.
Rajas											
Control											
		9.1	20	8.958333	15.42384	9.497515	5.799294	3.479504	2.809765	2.441756	0
		9.1	29.1	38.05833	53.48217	62.97969	68.77898	72.25848	75.06825	77.51001	77.51001
60	10										
		9.1	25.625	27.79436	7.656372	7.859209	4.313813	1.821298	0.820707	0.793154	0
		9.1	34.725	62.51936	70.17573	78.03494	82.34876	84.17005	84.99076	85.78391	85.78391
	15										
		9.1	25.375	31.17315	7.347352	8.354968	2.37421	2.212766	0.645296	1.096174	0
		9.1	34.475	65.64815	72.9955	81.35047	83.72468	85.93744	86.58274	87.67891	87.67891
	20										
		9.1	26.70833	34.10062	6.44924	6.841742	1.444588	2.359192	0.934385	0.86402	0
		9.1	35.80833	69.90896	76.3582	83.19994	84.64453	87.00372	87.93811	88.80213	88.80213
