# Supplementary material for: A peleg modeling of water absorption in cold plasma-treated Chickpea (Cicer arietinum L.) cultivars
Source: Sci Rep. 2023 May 15;13:7857. doi: 10.1038/s41598-023-33802-y (PMC10185522; doi:10.1038/s41598-023-33802-y)
Supplement: Supplementary file 3 — Supplementary Information 3. [file 41598_2023_33802_MOESM3_ESM.pdf]

Supplementary Table S3 K_1, K_2 and RMSE value (%) of plasma treated chickpea cultivars during soaking in distilled water

KRIPA	Control			
Kc		k1	k2	
M0	8.4	0.024	0.012	
time	mt(pre)	mt(Exp)	(Mte-Mtp/Mte)^2	RMSE
3	58.4	55.49518	0.002739862	0.084035
4	63.95556	62.3574	0.000656848	
5	67.92381	64.34682	0.003090164	
6	70.9	70.79126	2.35937E-06	
7	73.21481	74.87384	0.000490961	
8	75.06667	75.65387	6.02449E-05	
9	76.58182	75.65387	0.000150446	

KRIPA 40		10				15					20			
40-10		k1	k2		40-15		k1	k2		40-20		k1	k2	
M0	8.4	0.0206	0.0109		M0	8.4	0.0188	0.01		M0	8.4	0.0166	0.0101	RMSE
time	mt(pre)	mt(Exp)	(Mte-Mtp/Mte)^2	RMSE	time	mt(pre)	mt(Exp)	(Mte-Mtp/Mte)^2	RMSE	time	mt(pre)	mt(Exp)	(Mte-Mtp/Mte)^2	0.027552
3	64.68518	63.08472	0.000643633	0.034033	3	69.87541	68.59696	0.000347345	0.036052	3	72.36588	71.19492	0.000270516	
4	70.7053	70.69342	2.8225E-08		4	76.42721	76.80738	2.44987E-05		4	78.57544	77.9705	6.01943E-05	
5	74.9779	75.27411	1.54853E-05		5	81.07442	81.60065	4.15884E-05		5	82.91565	83.10102	4.9758E-06	
6	78.16744	79.76647	0.000401858		6	84.54213	84.83066	1.1568E-05		6	86.12021	85.99057	2.2727E-06	
7	80.63942	81.20671	4.8801E-05		7	87.22883	89.78656	0.000811495		7	88.58328	90.3384	0.000377459	
8	82.6115	82.90199	1.22777E-05		8	89.37166	89.92661	3.80834E-05		8	90.53552	90.53776	6.10375E-10	
9	84.2214	82.90199	0.000253297		9	91.12059	89.92661	0.000176285		9	92.12093	90.53776	0.00030577	

KRIPA 50		10				15					20			
50-10		k1	k2		50-15		k1	k2		50-20		k1	k2	
M0	8.4	0.0112	0.0107		M0	8.4	0.0109	0.01		M0	8.4	0.0108	0.0093	RMSE
time	mt(pre)	mt(Exp)	(Mte-Mtp/Mte)^2	RMSE	time	mt(pre)	mt(Exp)	(Mte-Mtp/Mte)^2	RMSE	time	mt(pre)	mt(Exp)	(Mte-Mtp/Mte)^2	0.037527
3	77.68406	73.71877	0.002893303	0.062264	3	81.74963	79.8961	0.000538207	0.027233	3	85.91938	83.83743	0.000616686	
4	82.47407	84.67999	0.000678603		4	86.98546	87.80831	8.78145E-05		4	91.73333	90.76572	0.000113647	
5	85.67975	87.06818	0.000254288		5	90.50181	90.09749	2.0138E-05		5	95.66003	96.44975	6.70403E-05	
6	87.9756	88.17293	5.0086E-06		6	93.02623	93.73058	5.6469E-05		6	98.49009	100.8627	0.00055333	
7	89.70081	90.11919	2.15532E-05		7	94.92658	95.35316	2.00145E-05		7	100.6266	101.1406	2.58268E-05	
8	91.04463	90.9606	8.53321E-07		8	96.4088	96.5361	1.73889E-06		8	102.2967	102.1587	1.82462E-06	
9	92.12093	90.9606	0.000162725		9	97.59722	96.5361	0.000120824		9	103.6381	102.1587	0.000209704	

KRIPA 60		10				15					20			
60-10		k1	k2		60-15		k1	k2		60-20		k1	k2	
M0	8.4	0.0102	0.011		M0	8.4	0.01	0.0095		M0	8.4	0.0098	0.0094	RMSE
time	mt(pre)	mt(Exp)	(Mte-Mtp/Mte)^2	RMSE	time	mt(pre)	mt(Exp)	(Mte-Mtp/Mte)^2	RMSE	time	mt(pre)	mt(Exp)	(Mte-Mtp/Mte)^2	0.047832
3	77.84444	73.91703	0.002823096	0.06359	3	86.32208	84.08272	0.000709307	0.045972	3	87.34737	84.61755	0.001040752	
4	82.20074	83.56312	0.000265809		4	91.73333	90.85719	9.29883E-05		4	92.78819	91.32561	0.000256479	
5	85.08712	87.34866	0.000670346		5	95.35652	97.38605	0.000434307		5	96.42817	97.99377	0.000255251	
6	87.14016	88.55124	0.000253931		6	97.95224	100.5618	0.000673415		6	99.03444	101.5424	0.000610041	
7	88.67523	89.00589	1.38015E-05		7	99.90327	101.2825	0.000185434		7	100.9926	101.84	6.92451E-05	
8	89.8664	89.8473	4.51785E-08		8	101.4233	101.5783	2.32906E-06		8	102.5176	102.0796	1.84123E-05	
9	90.81758	89.8473	0.000116624		9	102.6408	101.5783	0.000109422		9	103.739	102.0796	0.000264241	

VIRAT	Control			
VC		k1	k2	
M0	8.2	0.0322	0.0104	
time	mt(pre)	mt(Exp)	(Mte-Mtp/Mte)^2	RMSE
3	55.51861	52.85741	0.002534796	0.064599
4	62.40054	63.28669	0.00019606	
5	67.58242	69.26725	0.00059164	
6	71.62495	71.75961	3.52172E-06	
7	74.86667	76.96723	0.000744834	
8	77.52409	77.75223	8.60927E-06	
9	79.74213	77.75223	0.000654995	

VIRAT 40		10				15					20			
40-10		k1	k2		40-15		k1	k2		40-20		k1	k2	
M0	8.2	0.0075	0.0115		M0	8.2	0.005	0.0105		M0	8.2	0.0046	0.0104	
time	mt(pre)	mt(Exp)	(Mte-Mtp/Mte)^2	RMSE	time	mt(pre)	mt(Exp)	(Mte-Mtp/Mte)^2	RMSE	time	mt(pre)	mt(Exp)	(Mte-Mtp/Mte)^2	RMSE
3	79.62857	79.38479	9.42999E-06	0.012327	3	90.39178	92.22278	0.000394185	0.028972	3	91.99888	93.07176	0.000132882	0.026272
4	82.96636	82.46584	3.68373E-05		4	93.30638	93.91107	4.14592E-05		4	94.78009	95.18242	1.7867E-05	
5	85.12308	85.19488	7.10407E-07		5	95.15652	94.61971	3.21869E-05		5	96.53922	95.48344	0.000122262	
6	86.63137	85.78523	9.72892E-05		6	96.43529	95.14243	0.000184654		6	97.75224	96.04366	0.000316469	
7	87.74545	87.79738	3.49842E-07		7	97.37197	96.54243	7.38319E-05		7	98.63928	97.77466	7.81973E-05	
8	88.60201	88.68089	7.91087E-07		8	98.08764	99.12626	0.000109782		8	99.31617	99.78914	2.2464E-05	
9	89.28108	88.68089	4.58063E-05		9	98.65226	99.12626	2.28648E-05		9	99.84969	99.78914	3.68291E-07	

VIRAT 50		10				15					20			
50-10		k1	k2		50-15		k1	k2		50-20		k1	k2	
M0	8.2	0.0051	0.0104		M0	8.2	0.0045	0.0103		M0	8.2	0.0044	0.0102	
time	mt(pre)	mt(Exp)	(Mte-Mtp/Mte)^2	RMSE	time	mt(pre)	mt(Exp)	(Mte-Mtp/Mte)^2	RMSE	time	mt(pre)	mt(Exp)	(Mte-Mtp/Mte)^2	RMSE
3	90.84463	91.95882	0.000146803	0.023563	3	92.94576	91.75661	0.000167957	0.015965	3	93.91429	92.82049	0.000138863	0.014481
4	93.8531	93.81679	1.49826E-07		4	95.72735	95.70031	7.98741E-08		4	96.69558	96.73966	2.07655E-07	
5	95.76567	94.18161	0.000282889		5	97.48571	97.48398	3.17397E-10		5	98.45271	98.51266	3.7035E-07	
6	97.08889	96.05964	0.000114806		6	98.69774	98.25275	2.05116E-05		6	99.66341	99.27692	1.51566E-05	
7	98.05879	97.90304	2.53079E-06		7	99.58381	98.82005	5.97352E-05		7	100.5483	99.8409	5.01992E-05	
8	98.80023	99.05135	6.42776E-06		8	100.2598	100.1622	9.50301E-07		8	101.2233	101.1753	2.25013E-07	
9	99.38541	99.05135	1.13743E-05		9	100.7926	100.1622	3.96112E-05		9	101.7551	101.1753	3.28438E-05	

VIRAT 60		10				15					20			
60-10		k1	k2		60-15		k1	k2		60-20		k1	k2	
M0	8.2	0.0051	0.0104		M0	8.2	0.0045	0.0103		M0	8.2	0.0043	0.0102	
time	mt(pre)	mt(Exp)	(Mte-Mtp/Mte)^2	RMSE	time	mt(pre)	mt(Exp)	(Mte-Mtp/Mte)^2	RMSE	time	mt(pre)	mt(Exp)	(Mte-Mtp/Mte)^2	RMSE
3	90.84463	92.20302	0.000217051	0.022553	3	92.94576	92.0725	8.99565E-05	0.011628	3	94.15989	93.10172	0.000129178	0.013421
4	93.8531	94.05831	4.75973E-06		4	95.72735	95.95468	5.61266E-06		4	96.8918	97.15511	7.3452E-06	
5	95.76567	94.44285	0.000196186		5	97.48571	97.76812	8.34385E-06		5	98.61591	98.7479	1.7865E-06	
6	97.08889	96.31785	6.40828E-05		6	98.69774	98.55537	2.08667E-06		6	99.80305	99.53083	7.48049E-06	
7	98.05879	98.13855	6.60445E-07		7	99.58381	99.10214	2.36232E-05		7	100.6703	100.1135	3.09333E-05	
8	98.80023	99.30473	2.58099E-05		8	100.2598	100.4616	4.03449E-06		8	101.3315	101.4265	8.75737E-07	
9	99.38541	99.30473	6.60093E-07		9	100.7926	100.4616	1.08534E-05		9	101.8524	101.4265	1.76392E-05	

VISHAL	Control			
C		k1	k2	
M0	10	0.0318	0.011	
time	mt(pre)	mt(Exp)	(Mte-Mtp/Mte)^2	RMSE
3	56.2963	55.21435	0.000383981	0.047933
4	62.77045	63.73489	0.000228982	
5	67.60369	65.72934	0.000813169	
6	71.34969	72.18974	0.000135411	
7	74.33824	76.28181	0.000649172	
8	76.77796	77.06358	1.37364E-05	
9	78.80734	77.06358	0.000512005	

VISHAL 40		10		
40-10		k1	k2	
M0	10	0.0306	0.0097	
time	mt(pre)	mt(Exp)	(Mte-Mtp/Mte)^2	RMSE
3	60.25126	55.49388	0.007349269	0.102693
4	67.63689	68.91285	0.000342827	
5	73.21113	76.26225	0.001600664	
6	77.56757	80.16797	0.001052156	
7	81.06599	81.55056	3.53069E-05	
8	83.93715	83.61747	1.46168E-05	
9	86.33588	83.61747	0.001056905	

	15			
40-15		k1	k2	
M0	10	0.0234	0.0105	
time	mt(pre)	mt(Exp)	(Mte-Mtp/Mte)^2	RMSE
3	64.64481	61.07452	0.003417333	0.06995
4	71.16208	72.19723	0.000205574	
5	75.87615	77.78934	0.000604886	
6	79.44444	80.92685	0.000335545	
7	82.23942	83.58058	0.000257482	
8	84.4879	84.44228	2.91781E-07	
9	86.33588	84.44228	0.000502868	

	20			
40-20		k1	k2	
M0	10	0.0201	0.0108	
time	mt(pre)	mt(Exp)	(Mte-Mtp/Mte)^2	RMSE
3	67.14286	62.24358	0.006195477	0.09385
4	73.19115	74.72679	0.000422302	
5	77.47638	80.2849	0.001223729	
6	80.67138	82.98289	0.00077592	
7	83.14525	83.97866	9.84875E-05	
8	85.11737	84.7758	1.62333E-05	
9	86.72634	84.7758	0.000529377	

VISHAL 50		10				15					20			
50-10		k1	k2		50-15		k1	k2		50-20		k1	k2	
M0	10	0.0218	0.0106		M0	10	0.0206	0.0105		M0	10	0.0178	0.0106	
time	mt(pre)	mt(Exp)	(Mte-Mtp/Mte)^2	RMSE	time	mt(pre)	mt(Exp)	(Mte-Mtp/Mte)^2	RMSE	time	mt(pre)	mt(Exp)	(Mte-Mtp/Mte)^2	RMSE
3	65.97015	62.54657	0.002996076	0.078786	3	67.58157	65.1334	0.001412787	0.063377	3	70.48387	67.57741	0.001849809	0.059381
4	72.3053	72.07481	1.02266E-05		4	73.89776	72.52034	0.00036076		4	76.44518	76.1221	1.80138E-05	
5	76.84492	79.52061	0.001132174		5	78.39945	81.34111	0.00130787		5	80.62147	82.27001	0.00040153	
6	80.25761	83.70921	0.001700177		6	81.77033	84.13621	0.000790711		6	83.71007	86.50169	0.001041505	
7	82.91667	84.38275	0.000301864		7	84.38895	85.20293	9.12691E-05		7	86.08696	87.19884	0.00016259	
8	85.0469	84.96816	8.58891E-07		8	86.48184	86.52744	2.77738E-07		8	87.97271	87.85043	1.93725E-06	
9	86.79181	84.96816	0.000460649		9	88.19288	86.52744	0.000370467		9	89.5053	87.85043	0.000354844	

VISHAL 60		10				15					20			
60-10		k1	k2		60-15		k1	k2		60-20		k1	k2	
M0	10	0.0246	0.0094		M0	10	0.0126	0.0104		M0	10	0.0125	0.0104	
time	mt(pre)	mt(Exp)	(Mte-Mtp/Mte)^2	RMSE	time	mt(pre)	mt(Exp)	(Mte-Mtp/Mte)^2	RMSE	time	mt(pre)	mt(Exp)	(Mte-Mtp/Mte)^2	RMSE
3	66.81818	62.57273	0.004603378	0.08384	3	78.49315	80.34732	0.000532544	0.045857	3	78.64989	80.47191	0.000512651	0.045789
4	74.30868	75.06886	0.000102545		4	83.80074	80.70542	0.001470973		4	83.93715	80.82962	0.001478059	
5	79.8324	82.04294	0.000725961		5	87.39938	87.06135	1.50748E-05		5	87.51938	87.15619	1.73647E-05	
6	84.07407	87.18984	0.001277021		6	90	90.68895	5.77118E-05		6	90.10681	90.82265	6.21217E-05	
7	87.43363	88.69912	0.000203555		7	91.96721	92.00421	1.61661E-07		7	92.06331	92.09589	1.25184E-07	
8	90.16032	89.92476	6.86199E-06		8	93.50731	93.85162	1.34593E-05		8	93.59457	93.94183	1.36647E-05	
9	92.41758	89.92476	0.000768466		9	94.74576	93.85162	9.07677E-05		9	94.82564	93.94183	8.85108E-05	

VIJAY	Control			
C		k1	k2	
M0	10.1	0.0293	0.0113	
time	mt(pre)	mt(Exp)	(Mte-Mtp/Mte)^2	RMSE
3	57.56835	57.35586	1.37263E-05	0.045977
4	63.79128	64.45072	0.00010469	
5	68.37506	66.18222	0.001097822	
6	71.89197	72.64497	0.000107444	
7	74.67565	76.69965	0.000696363	
8	76.93375	77.50125	5.36188E-05	
9	78.80229	77.50125	0.000281814	

VIJAY 40		10		
40-10		k1	k2	
M0	10.1	0.0228	0.0108	
time	mt(pre)	mt(Exp)	(Mte-Mtp/Mte)^2	RMSE
3	64.44783	62.30389	0.001184111	0.039588
4	70.70606	70.98275	1.51941E-05	
5	75.20417	75.69839	4.26256E-05	
6	78.59315	79.90749	0.000270544	
7	81.23821	81.29728	5.27899E-07	
8	83.36007	83.56757	6.16536E-06	
9	85.1	83.56757	0.000336267	

	15			
40-15		k1	k2	
M0	10.1	0.0228	0.0108	
time	mt(pre)	mt(Exp)	(Mte-Mtp/Mte)^2	RMSE
3	64.44783	62.56937	0.000901322	0.042442
4	70.70606	70.31911	3.02807E-05	
5	75.20417	76.05388	0.000124824	
6	78.59315	80.57365	0.000604176	
7	81.23821	82.00068	8.64581E-05	
8	83.36007	83.59948	8.20121E-06	
9	85.1	83.59948	0.000322162	

	20			
40-20		k1	k2	
M0	10.1	0.0168	0.0113	
time	mt(pre)	mt(Exp)	(Mte-Mtp/Mte)^2	RMSE
3	69.2716	66.74531	0.001432601	0.049027
4	74.61613	74.93186	1.77543E-05	
5	78.31282	79.63262	0.000274682	
6	81.02199	82.80826	0.000465319	
7	83.0927	84.23047	0.00018246	
8	84.72687	84.83742	1.69803E-06	
9	86.04937	84.83742	0.000204078	

VIJAY 50		10				15					20			
50-10		k1	k2		50-15		k1	k2		50-20		k1	k2	
M0	10.1	0.0226	0.0108		M0	10.1	0.0225	0.0108		M0	10.1	0.0165	0.0112	
time	mt(pre)	mt(Exp)	(Mte-Mtp/Mte)^2	RMSE	time	mt(pre)	mt(Exp)	(Mte-Mtp/Mte)^2	RMSE	time	mt(pre)	mt(Exp)	(Mte-Mtp/Mte)^2	RMSE
3	64.64545	62.57036	0.001099861	0.039155	3	64.74481	63.04762	0.000724642	0.042628	3	69.98024	67.19116	0.001723046	0.047938
4	70.89027	71.22915	2.26342E-05		4	70.9828	70.76755	9.25192E-06		4	75.35285	75.34737	5.3066E-09	
5	75.37415	75.93475	5.45039E-05		5	75.45948	76.48183	0.000178685		5	79.06552	80.03201	0.000145838	
6	78.74989	80.1353	0.000298888		6	78.82852	80.98634	0.000709913		6	81.78459	83.19726	0.000288312	
7	81.3831	81.52238	2.91902E-06		7	81.45576	82.40875	0.000133731		7	83.86185	84.61495	7.9214E-05	
8	83.4945	83.7883	1.22959E-05		8	83.56189	84.00246	2.75071E-05		8	85.50057	85.22	1.08392E-05	
9	85.22521	83.7883	0.000294096		9	85.28797	84.00246	0.000234189		9	86.82634	85.22	0.0003553	

VIJAY 60		10				15					20			
60-10		k1	k2		60-15		k1	k2		60-20		k1	k2	
M0	10.1	0.0224	0.0108		M0	10.1	0.022	0.0108		M0	10.1	0.0127	0.0105	
time	mt(pre)	mt(Exp)	(Mte-Mtp/Mte)^2	RMSE	time	mt(pre)	mt(Exp)	(Mte-Mtp/Mte)^2	RMSE	time	mt(pre)	mt(Exp)	(Mte-Mtp/Mte)^2	RMSE
3	64.84453	62.75209	0.001111853	0.039232	3	65.24706	63.04762	0.001216989	0.043254	3	77.9733	80.19894	0.000770144	0.046654
4	71.07561	71.39755	2.03322E-05		4	71.44969	71.46003	2.09339E-08		4	83.22614	80.55785	0.001097115	
5	75.54503	76.09649	5.25172E-05		5	75.88947	76.49907	6.34992E-05		5	86.78712	86.90563	1.85981E-06	
6	78.90734	80.29135	0.000297125		6	79.22442	81.00357	0.000482409		6	89.36024	90.58365	0.000182409	
7	81.52857	81.67663	3.2859E-06		7	81.82131	82.42599	5.38164E-05		7	91.3065	91.86076	3.64064E-05	
8	83.62941	83.93965	1.36602E-05		8	83.90074	84.25344	1.75243E-05		8	92.83009	93.70224	8.66315E-05	
9	85.35084	83.93965	0.00028264		9	85.60336	84.25344	0.000256707		9	94.05522	93.70224	1.41912E-05	

DIGVIJAY	Control			
C		k1	k2	
M0	9.3	0.0294	0.0113	
time	mt(pre)	mt(Exp)	(Mte-Mtp/Mte)^2	RMSE
3	56.69336	56.42829	2.20666E-05	0.046499
4	62.9193	63.53079	9.26429E-05	
5	67.50722	65.26403	0.001181366	
6	71.0284	71.73316	9.65278E-05	
7	73.81613	75.7916	0.000679362	
8	76.07796	76.59392	4.53777E-05	
9	77.94989	76.59392	0.000313406	

DIGVIJAY 40		10					15					20		
40-10		k1	k2		40-15		k1	k2		40-20		k1	k2	
M0	9.3	0.0245	0.0111		M0	9.3	0.0236	0.0111		M0	9.3	0.0157	0.0111	
time	mt(pre)	mt(Exp)	(Mte-Mtp/Mte)^2	RMSE	time	mt(pre)	mt(Exp)	(Mte-Mtp/Mte)^2	RMSE	time	mt(pre)	mt(Exp)	(Mte-Mtp/Mte)^2	RMSE
3	61.20311	61.82827	0.000102235	0.042089	3	62.02408	62.8048	0.00015453	0.042706	3	70.52449	70.8399	1.98241E-05	0.033043
4	67.35515	66.61345	0.000123976		4	68.12353	67.55334	7.12446E-05		4	75.85574	74.38121	0.000392987	
5	71.8	69.73154	0.000879901		5	72.51113	70.64863	0.000694996		5	79.52472	78.73208	0.000101357	
6	75.16169	76.6125	0.000358608		6	75.81885	77.48078	0.000460087		6	82.20401	83.90487	0.000410927	
7	77.79315	78.9961	0.000231891		7	78.40168	79.84856	0.000328348		7	84.24647	85.24047	0.000135982	
8	79.909	80.43213	4.23018E-05		8	80.47438	81.27528	9.71056E-05		8	85.85502	86.14005	1.09486E-05	
9	81.64727	80.43213	0.000228239		9	82.17449	81.27528	0.000122407		9	87.15467	86.14005	0.000138739	

DIGVIJAY 50		10				15					20			
50-10		k1	k2		50-15		k1	k2		50-20		k1	k2	
M0	9.3	0.0128	0.0111		M0	9.3	0.0107	0.0112		M0	9.3	0.0104	0.0106	
time	mt(pre)	mt(Exp)	(Mte-Mtp/Mte)^2	RMSE	time	mt(pre)	mt(Exp)	(Mte-Mtp/Mte)^2	RMSE	time	mt(pre)	mt(Exp)	(Mte-Mtp/Mte)^2	RMSE
3	74.37592	70.31831	0.003329688	0.06813	3	77.02009	76.53538	4.01087E-05	0.016956	3	80.39005	76.33225	0.002825957	0.057844
4	79.23007	81.29422	0.000644708		4	81.37207	81.7011	1.6218E-05		4	85.05758	85.83366	8.17521E-05	
5	82.50644	84.29861	0.000451975		5	84.26252	84.78738	3.83202E-05		5	88.16435	89.6352	0.000269265	
6	84.86675	86.06569	0.000194059		6	86.32182	86.30541	3.61773E-08		6	90.38108	91.22842	8.62681E-05	
7	86.64807	86.73515	1.00797E-06		7	87.86341	86.90769	0.000120933		7	92.04232	92.34858	1.09983E-05	
8	88.04016	88.14828	1.50455E-06		8	89.06072	89.81851	7.11816E-05		8	93.33361	92.81182	3.16073E-05	
9	89.15803	88.14828	0.00013122		9	90.01749	89.81851	4.90778E-06		9	94.36616	92.81182	0.00028047	

DIGVIJAY 60		10				15					20			
60-10		k1	k2		60-15		k1	k2		60-20		k1	k2	
M0	9.3	0.0084	0.0116		M0	9.3	0.0073	0.0112		M0	9.3	0.0066	0.011	
time	mt(pre)	mt(Exp)	(Mte-Mtp/Mte)^2	RMSE	time	mt(pre)	mt(Exp)	(Mte-Mtp/Mte)^2	RMSE	time	mt(pre)	mt(Exp)	(Mte-Mtp/Mte)^2	RMSE
3	78.74444	77.73933	0.000167168	0.017981	3	82.64963	81.57778	0.000172635	0.018761	3	85.05758	83.25225	0.000470239	0.024655
4	82.2927	82.09608	5.73618E-06		4	86.07543	86.42844	1.66823E-05		4	88.35138	88.08595	9.07999E-06	
5	84.6012	85.50591	0.00011195		5	88.28894	89.10567	8.40133E-05		5	90.46883	91.4066	0.000105253	
6	86.22308	86.64732	2.3973E-05		6	89.83691	89.68974	2.69271E-06		6	91.94463	92.1026	2.942E-06	
7	87.425	87.42057	2.56612E-09		7	90.98028	90.31189	5.47743E-05		7	93.03206	93.15973	1.8782E-06	
8	88.35138	88.64413	1.09065E-05		8	91.85934	92.26984	1.97929E-05		8	93.8666	93.64253	5.72538E-06	
9	89.08723	88.64413	2.49868E-05		9	92.55624	92.26984	9.63468E-06		9	94.52727	93.64253	8.92662E-05	

RAJAS	Control			
C		k1	k2	
M0	9.1	0.0323	0.0107	
time	mt(pre)	mt(Exp)	(Mte-Mtp/Mte)^2	RMSE
3	55.68385	53.48217	0.001694692	0.050816
4	62.36232	62.97969	9.60921E-05	
5	67.37506	68.77898	0.000416652	
6	71.27617	72.25848	0.00018481	
7	74.39851	75.06825	7.95979E-05	
8	76.95411	77.51001	5.14357E-05	
9	79.08445	77.51001	0.000412609	

RAJAS 40		10				15					20			
40-10		k1	k2		40-15		k1	k2		40-20		k1	k2	
M0	9.1	0.0323	0.0104		M0	9.1	0.0239	0.011		M0	9.1	0.0124	0.0112	
time	mt(pre)	mt(Exp)	(Mte-Mtp/Mte)^2	RMSE	time	mt(pre)	mt(Exp)	(Mte-Mtp/Mte)^2	RMSE	time	mt(pre)	mt(Exp)	(Mte-Mtp/Mte)^2	RMSE
3	56.34409	53.64172	0.002537961	0.064073	3	61.82408	60.04473	0.000878154	0.038191	3	75.1793	72.47773	0.001389378	0.04189
4	63.2272	64.08825	0.000180509		4	68.01016	67.83525	6.64876E-06		4	80.02199	79.30245	8.23259E-05	
5	68.41198	70.07777	0.000565039		5	72.47136	73.10477	7.50722E-05		5	83.28398	82.9115	2.01819E-05	
6	72.45797	72.57365	2.54048E-06		6	75.84082	76.7787	0.000149216		6	85.63061	85.50778	2.06351E-06	
7	75.70324	77.78844	0.000718568		7	78.47562	79.83518	0.000290008		7	87.39978	87.04722	1.64043E-05	
8	78.36407	78.57447	7.17002E-06		8	80.5924	80.71633	2.35742E-06		8	88.78127	87.69104	0.000154572	
9	80.58531	78.57447	0.000654925		9	82.33027	80.71633	0.000399805		9	89.88995	87.69104	0.000628788	

RAJAS 50		10				15					20		
50-10		k1	k2		50-15		k1	k2		50-20		k1	k2	
M0	9.1	0.0131	0.0115		M0	9.1	0.013	0.0113		M0	9.1	0.0097	0.0115	
time	mt(pre)	mt(Exp)	(Mte-Mtp/Mte)^2	RMSE	time	mt(pre)	mt(Exp)	(Mte-Mtp/Mte)^2	RMSE	time	mt(pre)	mt(Exp)	(Mte-Mtp/Mte)^2	RMSE
3	72.12521	69.10167	0.001914491	0.056666	3	73.06588	70.03134	0.001877591	0.05402	3	76.9733	74.69383	0.000931323	0.036414
4	76.7819	77.75716	0.000157312		4	77.82852	78.7207	0.000128447		4	80.91329	81.65157	8.17554E-05	
5	79.92153	82.00867	0.000647713		5	81.04245	82.76245	0.000431909		5	83.50476	84.22743	7.36148E-05	
6	82.18161	83.89748	0.000418287		6	83.35743	85.11037	0.0004242		6	85.33888	86.56835	0.000201704	
7	83.88632	84.48733	5.06027E-05		7	85.10434	85.61089	3.50092E-05		7	86.70532	87.15059	2.61038E-05	
8	85.21798	85.49257	1.03155E-05		8	86.46944	86.68995	6.47041E-06		8	87.76273	87.87418	1.60844E-06	
9	86.28696	85.49257	8.63416E-05		9	87.56556	86.68995	0.00010202		9	88.6053	87.87418	6.92239E-05	

RAJAS 60		10					15					20		
60-10		k1	k2		60-15		k1	k2		60-20		k1	k2	
M0	9.1	0.0125	0.0115		M0	9.1	0.0108	0.0114		M0	9.1	0.0091	0.0114	
time	mt(pre)	mt(Exp)	(Mte-Mtp/Mte)^2	RMSE	time	mt(pre)	mt(Exp)	(Mte-Mtp/Mte)^2	RMSE	time	mt(pre)	mt(Exp)	(Mte-Mtp/Mte)^2	RMSE
3	72.92979	70.17573	0.001540178	0.049483	3	75.76667	72.9955	0.00144123	0.044684	3	78.38406	76.3582	0.000703899	0.02981
4	77.47607	78.03494	5.1292E-05		4	80.02199	81.35047	0.00026668		4	82.22614	83.19994	0.000136991	
5	80.52857	82.34876	0.000488559		5	82.84631	83.72468	0.000110064		5	84.74297	84.64453	1.35246E-06	
6	82.71963	84.17005	0.000296943		6	84.85758	85.93744	0.000157898		6	86.51935	87.00372	3.09935E-05	
7	84.36882	84.99076	5.35499E-05		7	86.36269	86.58274	6.45902E-06		7	87.84016	87.93811	1.24061E-06	
8	85.65502	85.78391	2.25753E-06		8	87.53137	87.67891	2.83162E-06		8	88.86072	88.80213	4.35352E-07	
9	86.68621	85.78391	0.000110632		9	88.46508	87.67891	8.03967E-05		9	89.67296	88.80213	9.61675E-05	
